# β-HPV 5 and 8 E6 Disrupt Homology Dependent Double Strand Break Repair by Attenuating BRCA1 and BRCA2 Expression and Foci Formation

**DOI:** 10.1371/journal.ppat.1004687

**Published:** 2015-03-24

**Authors:** Nicholas A. Wallace, Kristin Robinson, Heather L. Howie, Denise A. Galloway

**Affiliations:** 1 Division of Human Biology, Fred Hutchinson Cancer Research Center, Seattle, Washington, United States of America; 2 Puget Sound Blood Center Research Institute, Seattle, Washington, United States of America; National Institute of Allergy and Infectious Diseases, National Institutes of Health, UNITED STATES

## Abstract

Recent work has explored a putative role for the E6 protein from some β-human papillomavirus genus (β-HPVs) in the development of non-melanoma skin cancers, specifically β-HPV 5 and 8 E6. Because these viruses are not required for tumor maintenance, they are hypothesized to act as co-factors that enhance the mutagenic capacity of UV-exposure by disrupting the repair of the resulting DNA damage. Supporting this proposal, we have previously demonstrated that UV damage signaling is hindered by β-HPV 5 and 8 E6 resulting in an increase in both thymine dimers and UV-induced double strand breaks (DSBs). Here we show that β-HPV 5 and 8 E6 further disrupt the repair of these DSBs and provide a mechanism for this attenuation. By binding and destabilizing a histone acetyltransferase, p300, β-HPV 5 and 8 E6 reduce the enrichment of the transcription factor at the promoter of two genes critical to the homology dependent repair of DSBs (BRCA1 and BRCA2). The resulting diminished BRCA1/2 transcription not only leads to lower protein levels but also curtails the ability of these proteins to form repair foci at DSBs. Using a GFP-based reporter, we confirm that this reduced foci formation leads to significantly diminished homology dependent repair of DSBs. By deleting the p300 binding domain of β-HPV 8 E6, we demonstrate that the loss of robust repair is dependent on viral-mediated degradation of p300 and confirm this observation using a combination of p300 mutants that are β-HPV 8 E6 destabilization resistant and p300 knock-out cells. In conclusion, this work establishes an expanded ability of β-HPV 5 and 8 E6 to attenuate UV damage repair, thus adding further support to the hypothesis that β-HPV infections play a role in skin cancer development by increasing the oncogenic potential of UV exposure.

## Author Summary

Human Papillomaviruses are a family of viruses with over 100 different members that infect mucous membranes and skin. Infections with some of these viruses are linked to cancers of the cervix and oropharynx. In this work, we explore the question of whether other members of this virus family may also contribute to skin cancer by inhibiting the ability of cells to repair the damage caused from UV exposure. Here, we build on our previous work showing that the E6 protein from two of these viruses (β-HPV 5 and 8) reduces the cellular response to UV damage by decreasing the abundance of two cellular proteins (p300 and ATR) involved in repairing the UV-damaged DNA, leading to more double strand DNA breaks following UV exposure. Here we show that the loss of p300 has further deleterious consequences, specifically that it results in diminished expression of two proteins (BRCA1 and BRCA2) involved in the repair of double strand breaks. Our data shows that this results in fewer BRCA1 and BRCA2 repair foci forming at sites of damage and ultimately in attenuated repair of these lesions. Together, this work provides further support for a link between β-HPV infections and skin cancer.

## Introduction

Human papillomaviruses (HPVs) are a large family of small double stranded DNA viruses that infect the mucosal and cutaneous epithelia of humans. Based on sequence homology, HPVs are divided into five genera [1]. Members of two of these genera, high risk α-HPV (HR-α HPVs) and some β-HPVs are associated with cancers [2–9]. The HR-α HPVs are the most well studied HPVs due to their well-known association with cancers of the anogenital track as well as of the oropharnyx [2,10]. Some β-HPVs have been connected to non-melanoma skin cancers (NMSC), leading to an increased interest in this genus of HPVs [8].

Although members of both the α and β genera of HPVs are associated with tumorigenesis, the details of these associations are markedly different. HR-α HPV genomes persist throughout the course of tumor development [11], resulting in tumors that are dependent on viral proteins [12–15]. β-HPV infections are far more transient, even when associated with tumors [7,9]. Therefore, unlike HR-α HPV associated tumors, the NMSCs that are linked to β-HPV infections do not require continual viral protein expression. Because of these differences, the proposed role of HPV in each of these types of tumors also varies. The functions of the two primary HR-α HPV oncogenes, HPV E6 and E7, are well characterized [16,17] and include degradation of p53 as well as pRB, and activation of telomerase [18–27]. Conversely, β-HPV proteins do not degrade p53 and only weakly activate telomerase [28–30]. Instead, β-HPV infections are believed to destabilize their host cell’s genome, increasing the probability of a mutation that can drive tumorigenesis independently of the viral genome.

Specifically, β-HPV infections are believed to contribute to NMSCs by disrupting UV-damaged repair and increasing the likelihood of UV-induced oncogenic mutations. In support of this hypothesis, our group and others have shown that expression of the E6 protein from some β-HPVs (β-HPV 5 and 8 E6) can attenuate UV-damage repair [29,31]. Mechanistically, the ability of these β-HPV E6 proteins to bind and destabilize the cellular histone acetyltransferase, p300, reduces the expression of the kinase responsible for initiating repair of UV-damaged DNA, Ataxia Telangiectasia and Rad3 related (ATR) [29,32,33]. The diminished abundance of ATR delays the repair of UVB-damaged DNA and diminishes G1 arrest [29]. Together, lack of robust repair and failure to halt cell cycle combine to increase the number of UVB-induced double strand breaks (DSBs), as replication forks more often encounter DNA crosslinks and collapse [29].

In addition to occurring more often, these DSBs also appear to be more persistent in cells expressing β-HPV 5 and 8 E6 proteins suggesting suboptimal DSB repair [29]. DSB repair can largely be divided into two categories based on whether or not it utilizes sequence homology [34–36]. Non-homologous end joining involves direct ligation of the two DNA ends and occurs without requiring sequence homology [37]. Homology dependent repair (HDR) begins with the resection of one of the DNA strands to generate a single strand segment of DNA (ssDNA) that is used to find homologous sequence to act as a template for the lesion’s repair [38].

BRCA1 is a central protein in the HDR pathway. Because p300 regulates BRCA1 expression at the transcriptional level by binding at the BRCA1 promoter, acetylating nearby histones promoting binding of the E2F1 transcription factor to the BRCA1 promoter [39,40], we hypothesized that the destabilization of p300 by β-HPV 5 and 8 E6 would reduce BRCA1 expression and inhibit HDR. Once activated, BRCA1 in combination with multiple other repair proteins promotes resection [41,42]. The resulting ssDNA is then rapidly coated with a complex of RPA proteins, including RPA70 [43,44]. Then, BRCA1 acts a second time in the pathway, facilitating RPA:RAD51 exchange in combination with PALB2 and BRCA2 [45,46]. Once loaded onto ssDNA, RAD51 facilitates strand invasion, a search for homologous sequence and resolution of DSB [47–49]. To test our hypothesis, we compared HDR in control and HPV 5 and 8 E6 expressing cells. Here, we show that β-HPV 5 and 8 E6 decrease expression and activation of both BRCA1 and BRCA2 leading to a delay in DSB repair, poor RAD51 foci resolution and an increased sensitivity to DSBs. Ultimately, by measuring HDR with a GFP-based HDR assay [50], we determined that these alterations in repair foci kinetics result in a reduced HDR.

As predicted by our hypothesis, these phenotypes are lost when the p300 binding domain of β-HPV 8 E6 is mutated. Further, we show that targeted knock out of p300 replicated this attenuation of repair. Finally, repair was rescued in β-HPV 5 and 8 E6 expressing cells by exogenous expression of a mutated p300 that is resistant to viral mediated destabilization. Together our work further supports a role for β-HPV infections as a cofactor in NMSC development by disrupting the repair of damaged DNA. Moreover, the data presented here expand the understanding of HDR as they demonstrate the requirement of p300 for robust HDR and that both DNA end resection and RAD51:RPA exchange can occur with limited BRCA1 or BRCA2 foci formation.

## Results

### β-HPV E6 expression attenuates DSB repair

**Fig 1:**
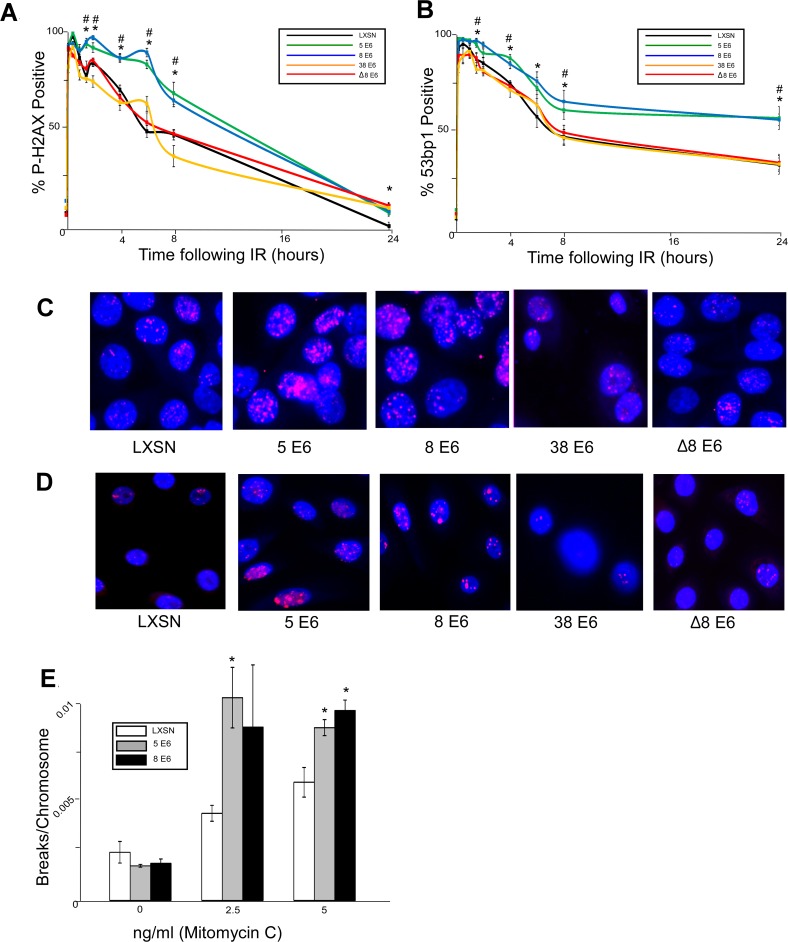
β-HPV 5 and 8 E6 attenuate double strand break repair. HFK cells were exposed to 4 gray of ionizing radiation and immunofluorescence microscopy was used to determine the percentage of cells that were positive for either (A.) p-H2AX foci or (B.) 53bp1 foci over a 24 hour period following irradiation. n ≥ 5. (C.) Representative images of cells stained for p-H2AX (pink) and nuclei (blue) 6 hours after exposure to IR. (D.) Representative images of cells stained for 53bp1 (pink) and nuclei (blue) 24 hours after exposure to IR. (E.) Chart depicts the mean number of breaks per chromosome measured in control cells as well as cells exposed to a DNA damaging agent. n = 3. Error bars depict the standard error of the mean. * denotes a significant difference of both β-HPV 5 and 8 E6 from vector control (LXSN) cells as determined by student t-test with a p value ≤ 0.05. # denotes that β-HPV Δ8 E6 was significantly different from corresponding wt β-HPV 8 E6 with a p-value ≤ 0.05 from a student T-test.

To investigate the potential for β-HPV 5 and 8 E6 expression to disrupt DSB repair, vector control, β-HPV 5 and 8 E6 expressing HFKs were generated. HPV E6 expression was confirmed by rtPCR (S1 Fig.). DSBs were induced by UVB exposure in these cells. Foci formation of the DSB repair protein, BRCA1, demonstrated similar frequency of these repair foci in each cell line (S2 Fig.) despite the fact that UVB induced DSBs occurs twice as often in cells expressing HPV 5 or 8 E6 [29]. Correcting for the difference in DSB abundance suggests that DSB repair was occurring inefficiently in HPV 5 and 8 E6 expressing keratinocytes. Because HPV 5 and 8 E6 alter the frequency of UVB-induced DSBs, ionizing radiation (IR) was used as an alternative for DSBs induction as IR induces DSBs directly and with equal frequency in all exposed cells. These cells were exposed to 4 gray of ionizing radiation (IR) and DSB repair was evaluated using immunofluorescence microscopy against two well characterized markers of DSBs, p-H2AX and 53bp1 foci [51,52]. Compared with the vector control, β-HPV 5 and 8 E6 expression did not alter the rapid initial kinetics of either p-H2AX or 53bp1 foci formation (Fig. 1). However, the efficient resolution of these damage foci was significantly delayed by the expression of either β-HPV 5 or 8 E6 proteins over a 24 hour time course following IR exposure (Fig. 1). DSB markers remain significantly elevated in cells expressing β-HPV 5 and 8 E6 a day after being exposed to IR. The attenuation of DSB repair by HPV 8 E6 was also assessed when co-expressed with the other primary viral oncogene HPV 8 E7. Co-expression of HPV 8 E7 did not inhibit the ability of HPV 8 E6 to impair repair of these lesions, but this data does not exclude the potential for HPV 8 E7 to independently attenuate DNA damage repair (S3 Fig.).

Because inhibition of UV-damage repair by β-HPV 5 and 8 E6 requires an intact p300 binding domain, the domain may also be important for their attenuation of DSB repair. To test this, HFKs stably expressing a previously characterized mutant of HPV 8 E6 (HPV Δ8 E6) with an inactivating mutation in its p300 binding domain were generated along with a line of keratinocytes expressing 38 E6, a β-HPV E6 that only binds p300 weakly resulting in a corresponding intermediate disruption of UV-damage repair [32,53]. HPV Δ8 and 38 E6 expression in these cells was confirmed by rtPCR (S1 Fig.). In contrast to wt HPV 8 E6, HPV Δ8 and 38 E6 did not significantly alter the repair of DSBs as determined by the resolution of both p-H2AX and 53bp1 foci following exposure to IR (Fig. 1). Importantly, this mutant maintains other functions associated with HPV 8 E6 according to results from our group and others [30,53].

Because assessing p-H2AX nor 53bp1 foci formation provides an indirect measure of DSBs, we performed chromosome fragility assays to confirm the ability of HPV 5 and 8 E6 to attenuate DSB repair. An approximately equal number of chromosome breaks were observed in untreated control as well as HPV 5 and 8 E6 expressing keratinocytes (Fig. 1E). However, expression of HPV 5 and 8 E6 significantly increased the number of DSBs in a dose dependent response to a DNA damaging agent (Fig. 1E).

### β-HPV 5 and 8 E6 expression attenuate BRCA1/2 expression and foci formation in response to DSBs

The most parsimonious connection between p300 and DSB repair is BRCA1, a protein centrally involved in the HDR pathway, as p300 is required for robust BRCA1 transcription [39,40]. We confirmed the previous reports of a role for p300 in the transcription of BRCA1 using Q-rtPCR of wt and p300 knockout HCT118 cells (S4A Fig.). As predicted by their ability to destabilize cellular p300 protein, HPV 5 and 8 E6 significantly reduce BRCA1 mRNA in HFKs (S4A Fig.). In support of a role for p300 in BRCA1 transcription, we also used chromatin immunoprecipitation (ChIP) to demonstrate that p300 is highly enriched at the BRCA1 promoter (S4B Fig.). Again, as we would anticipate, HPV 5 and 8 E6 expressing cells had significantly less p300 bound to the BRCA1 promoter (S4B Fig.).

**Fig 2:**
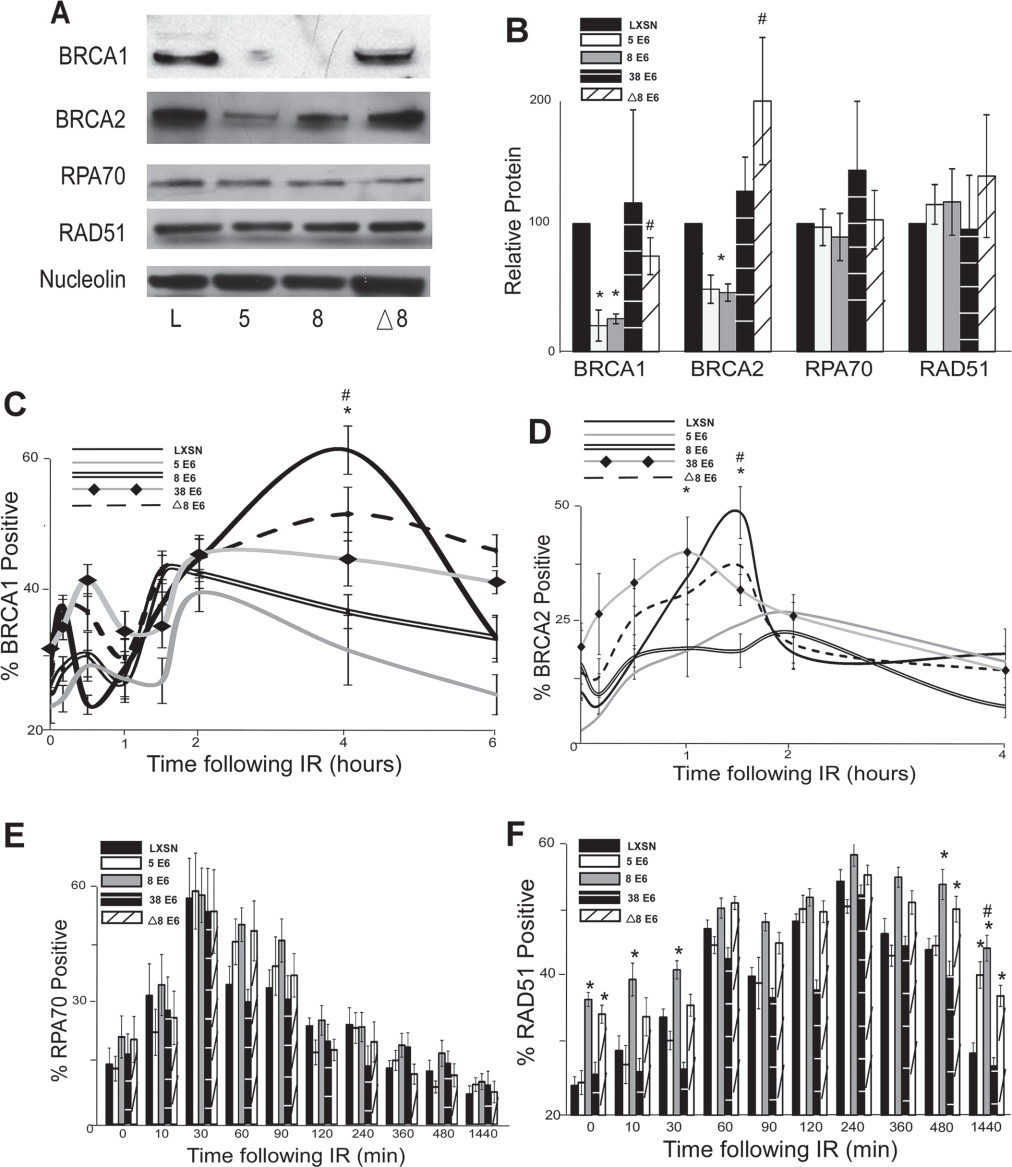
β-HPV 5 and 8 E6 attenuate HDR protein activation, abundance and resolution of damage-induced nuclear foci. (A.) Representative immunoblot showing BRCA1, BRCA2, RPA70 and RAD51 protein levels in HFK cells. Nucleolin is shown as a loading control. (B.) Densitometry of immunoblots (n = 3) of BRCA1, BRCA2, RPA70 and RAD51. The amount of protein in each sample was normalized to the corresponding amount of nucleolin and then data was set relative to vector control (LXSN) cells. L denotes vector control (LXSN) HFKs. 5 denotes HPV 5 E6 expressing HFKs. 8 denotes HPV 8 E6 expressing HFKs. (D.)-(G.) HFK cells were exposed to 4 gray of ionizing radiation and immunofluorescence microscopy was used to determine the percentage of cells positive for (C.) BRCA1, (D.) BRCA 2, (E.) RPA70 and (F.) RAD51 foci over a 6–24 hour period. Error bars represent standard errors of the mean. * denotes that both HPV 5 and 8 E6 are significantly different from vector control cells as determined by student T-test (p value ≤ 0.05). # denotes that β-HPV Δ8 E6 was significantly different from corresponding wt β-HPV 8 E6 with a p-value ≤ 0.05 from a student T-test.

To determine if β-HPV 5 and 8 E6 expression also decreased the abundance of BRCA1 protein, BRCA1 protein levels were measured by immunoblot. There was an approximately 75% reduction in BRCA1 protein levels in cells expressing β-HPV 5 or 8 E6 compared to controls (Fig. 2A-B). To determine whether other proteins in the HDR pathway were diminished, the abundance of three other proteins (BRCA2, RAD51 and RPA70) in the pathway were measured. Of these proteins, only BRCA2 was reduced by β-HPV 5 and 8 E6 expression (Fig. 2 A-B). ChIP and Q-rtPCR analysis confirmed a role for p300 in BRCA2 transcription and demonstrated that HPV 5 and 8 E6 expression reduced both enrichment of p300 at the BRCA2 promoter as well as the abundance of BRCA2 mRNA (S4C-D Fig.).

The proposed mechanism for β-HPV 5 and 8 E6-mediated reduction of BRCA1 and BRCA2 suggests that the ability to attenuate the expression of these proteins would be dependent on the binding and destabilization of p300 by β-HPV 5 and 8 E6. To test this, the abundance of these proteins was measured by immunoblot in β-HPV 8, Δ8 and 38 E6 expressing HFKs. Supporting this hypothesis, mutation of the p300 binding site abolished the ability of β-HPV 8 E6 to reduce steady state levels of both BRCA1 and BRCA2 (Fig. 2A-B). Further, expression of β-HPV 38 E6 that weakly binds p300 also did not reduce BRCA1 or BRCA2 expression (Fig. 2B and S5 Fig.). Indeed, HPV Δ8 E6 expression also failed to reduce BRCA1 and 2 mRNA levels (S4A,C Fig.).

In response to DSBs, many HDR proteins form distinct nuclear foci at sites of DSBs [41,43–45,49,54,55]. Once each protein completes its role in HDR, its nuclear foci are resolved. To determine if β-HPV 5 and 8 E6 hinder HDR, foci formation and resolution of HDR proteins was examined by immunofluorescence microscopy following IR exposure. Because β-HPV 5 and 8 E6 reduced BRCA1 and BRCA2 levels, the ability of these proteins to form and resolve nuclear foci in response to IR was examined. In control cells, BRCA1 foci formation peaked with about 60 percent of the observed cells containing foci after IR. Significantly fewer β-HPV 5 and 8 E6 expressing HFKs formed BRCA1 foci, and those BRCA1 positive cells had fewer foci per cell (Fig. 2C and S6–S7 Fig.). There was no notable difference in the resolution of BRCA1 foci in these cells (Fig. 2C). A similar percentage of control cells were positive for BRCA2 foci as were positive for BRCA1 foci (Fig. 2D and S8 Fig.). β-HPV 5 and 8 E6 expression reduced BRCA2 foci formation to half the levels seen in control cells as well as significantly reduced the abundance of foci in BRCA2 positive cells (Fig. 2D and S7 Fig.). As was the case with BRCA1 foci resolution, there was no significant difference in the rate of BRCA2 foci resolution in these cell lines (Fig. 2D).

Because BRCA1 promotes resection that rapidly results in RPA coated ssDNA, β-HPV 5 and 8 E6 expression may hinder RPA70 foci formation despite not directly effecting RPA70 protein levels. To evaluate this possibility, the kinetics of the RPA protein complex formation/resolution were determined by assaying for RPA70 foci. There were no significant differences in RPA 70 foci formation or resolution between control, β-HPV 5 and 8 E6 expressing cells (Fig. 2E and S9 Fig.). In line with formation of BRCA1 and BRCA2 foci formation, at peak, approximately 60% of the cells were RPA70 foci positive.

BRCA1 acts a second time in the HDR pathway downstream of RPA, when along with BRCA2, it catalyzes RPA:RAD51 exchange. To determine if reduced BRCA1/2 abundance and activation altered RPA:RAD51 exchange, RAD51 foci were measured by IF microscopy following IR. As expected by similar RPA70 resolution, the formation of RAD51 foci among our cell lines was not altered by β-HPV 5 or 8 E6 expression (Fig. 2F and S10 Fig.). Further consistent with previous data (Fig. 2C-E), at peak approximately 60% of the cells were positive for RAD51 foci (Fig. 2F and S10 Fig.). However, RAD51 foci resolution was significantly delayed by the expression of either HPV 5 or 8 E6 (Fig. 2F and S10 Fig.).

The data suggest that the alteration of HDR foci by β-HPV 5 and 8 E6 is dependent on the viral protein’s p300 binding domain. To evaluate this possibility, BRCA1, BRCA2, RPA70 and RAD51 foci formation/resolution was in HFK cells expressing HPV Δ8 or 38 E6. Significantly more HFK cells expressing HPV Δ8 E6 were BRCA1 foci positive following IR than HFK cells expressing β-HPV 8 E6. Cells expressing HPV 38 E6 had a moderate reduction of BRCA1 foci that correlates with their reported weak binding of p300 (Fig. 2C, [32]). A similar increase in BRCA2 foci formation was also observed with the introduction of this mutation and cell expressing and HPV 38 E6 displayed a mild reduction of BRCA2 foci (Fig. 2D and S8 Fig.). Further, although RPA70 foci formation was not changed, compared with HPV 8 E6 expressing cells, RAD51 foci were significantly less persistent in β-HPV Δ8 and 38 E6 expressing cells (Fig. 2E-F and S9–S10 Figs.).

Importantly the kinetics of foci formation for the four repair proteins largely fits the canonical HDR pathway. RPA foci form rapidly before subsiding as RAD51 foci reach their maxima. This exchange occurs at approximately the same time that both BRCA1 and BRCA2 foci are reaching their peak. The timing of BRCA1 and BRCA2 with respect to each other is more complex, however. There is an initial diminutive BRCA1 peak followed by a much larger secondary peak. We cannot determine whether these two peaks are the result of an asynchronous population or whether they represent the two roles of BRCA1 during HDR. However, BRCA2 foci peak between the two BRCA1 maxima supporting the latter interpretation.

### β-HPV 5 and 8 E6 expression attenuate homology dependent repair

**Fig 3:**
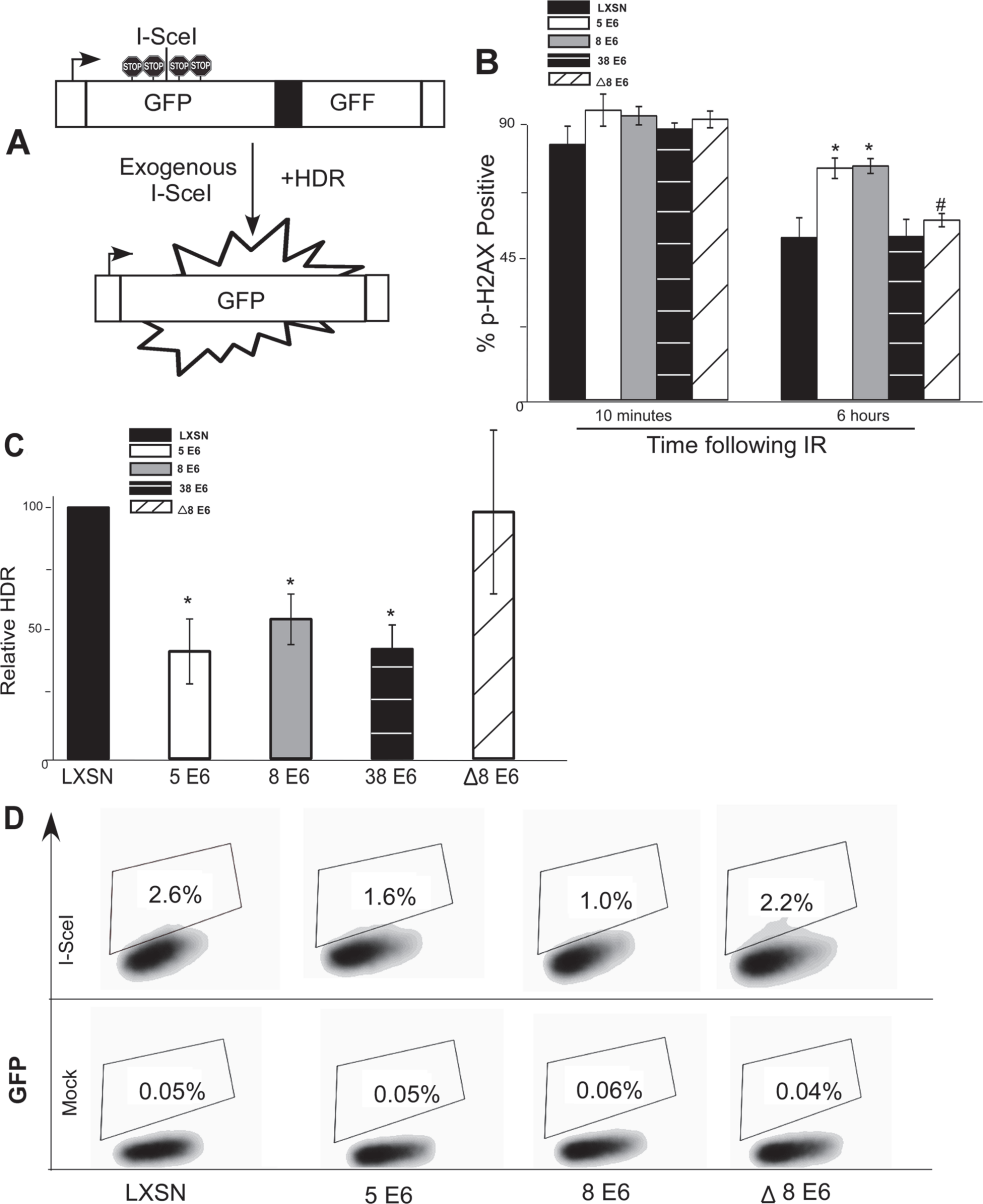
β-HPV 5 and 8 E6 attenuate homology dependent repair of double strand breaks. (A.) A representation of the DR-GFP cassette that is clonally integrated into U2OS cells. Briefly, the cassette consists of a promoter depicted here as a box with an arrow pointing to the right. Further, it contains two copies of a GFP gene (box labeled GFP). The first is a full copy of the gene interrupted by the recognition site for the rare cutting endonuclease, I-SceI, that is flanked by stop codons preventing read-through expression of GFP. The second copy of GFP is missing 5’ and 3’ sequence. Exogenous expression of I-SceI in these cells will introduce a double strand break in the first GFP gene that if repaired by homology dependent repair (labeled HDR) using the second gene as a template will result in a functional GFP gene. (B.) Immunofluorescence microscopy was used to measure p-H2AX foci persistence in U2OS DR-GFP cells following exposure to 4 gray of ionizing radiation. This chart depicts the percentage of p-H2AX positive cells 10 minutes and 6 hours after irradiation. (C.) This chart depicts the relative amount of homology dependent repair as measured by GFP positive cells following exogenous expression of I-SceI. Percentage of GFP positive β-HPV 5 and 8 E6 cells are given relative to the level of GFP positive vector control (LXSN) cells that were set to 100. (D.) Representative samples of FACS profiles used to generate data shown in (C.) Error Bars represent standard errors of the mean. n ≥ 3 for all experiments. * denotes significant difference from similarly treated control cells with a p-value ≤ 0.05 as determined by student T-test. # denotes that β-HPV Δ8 E6 was significantly different from corresponding wt β-HPV 8 E6 with a p-value ≤ 0.05 from a student T-test.

The attenuation of HDR protein expression and alteration of foci formation/resolution by β-HPV 5 and 8 E6 suggests that these viral proteins are inhibiting HDR of DSBs. The gold standard for measuring HDR utilizes the DR-GFP cassette clonally integrated into U2OS cells (U2OS DR-GFP) [50]. The cassette consists of two non-functional GFP genes separated by a spacer. The first gene is interrupted by an I-SceI recognition site and in frame stop codons preventing GFP expression. The second lacks 5’ and 3’ portions of the GFP gene (Fig. 3A). Exogenous expression of the I-SceI gene results in a DSB within the first GFP gene that when repaired using the second GFP gene as a template for HDR results in expression of a fully functional GFP.

After stably expressing β-HPV 5 and 8 E6 in the U2OS DR-GFP cells, the ability of β-HPV 5 and 8 E6 to attenuate DSB repair in these U2OS cells was confirmed by measuring p-H2AX foci resolution following exposure to IR. As in HFKs, p-H2AX foci were also more persistent in U2OS DR-GFP cells expressing β-HPV 5 or 8 E6 than vector control U2OS DR-GFP cells (Fig. 3B and S11 Fig.). Further confirming that HPV 5 and 8 E6 attenuated DSB repair similarly in U2OS and keratinocytes, BRCA1 and BRCA2 expression and foci formation in response to IR were reduced by HPV 5 and 8 E6 in U2OS cells (S12–S13 Fig.). Following transient transfection with an I-SceI expression vector, GFP positive cells were seen in all cell lines demonstrating the potential for HDR in all three cell lines. However, compared to vector control U2OS DR-GFP cells, β-HPV 5 and 8 E6 expressing U2OS DR-GFP cells were significantly less likely to repair the I-SceI induced DSB repair by HDR (Fig. 3C-D).

To determine if reduction of HDR efficiency by β-HPV 5 and 8 E6 was dependent on the viral proteins’ p300 binding domain, U2OS DR-GFP cells stably expressing β-HPV Δ8 E6 were generated. The ability of this mutation to prevent β-HPV 8 E6 from disrupting DSB repair, the kinetics of p-H2AX foci formation and resolution in these cells was observed. As expected, compared with the control, expression of HPV Δ8 E6 did not alter p-H2AX foci formation or resolution and the frequency of HDR was not significantly altered in U2OS DR-GFP cells expressing HPV Δ8 E6 (Fig. 3B-D and S11 Fig.).

As a comparison, we stably expressed HPV 38 E6 in U2OS DR-GFP cells and measured the frequency of HDR as well as persistence of DSBs following IR. In these assays, HPV 38 E6 exhibited an intermediate phenotype. While HPV 38 E6 expressing U2OS cells did not have increased p-H2AX foci persistence (Fig. 3B), the frequency of HDR in these cells was significantly reduced (Fig. 3C and S14 Fig.). This is in line with our previous reports that depending on the assay, the binding of p300 by HPV 38 E6 is adequate to disrupt some DDR signaling processes [29].

### p300 is required for efficient DSB repair

**Fig 4:**
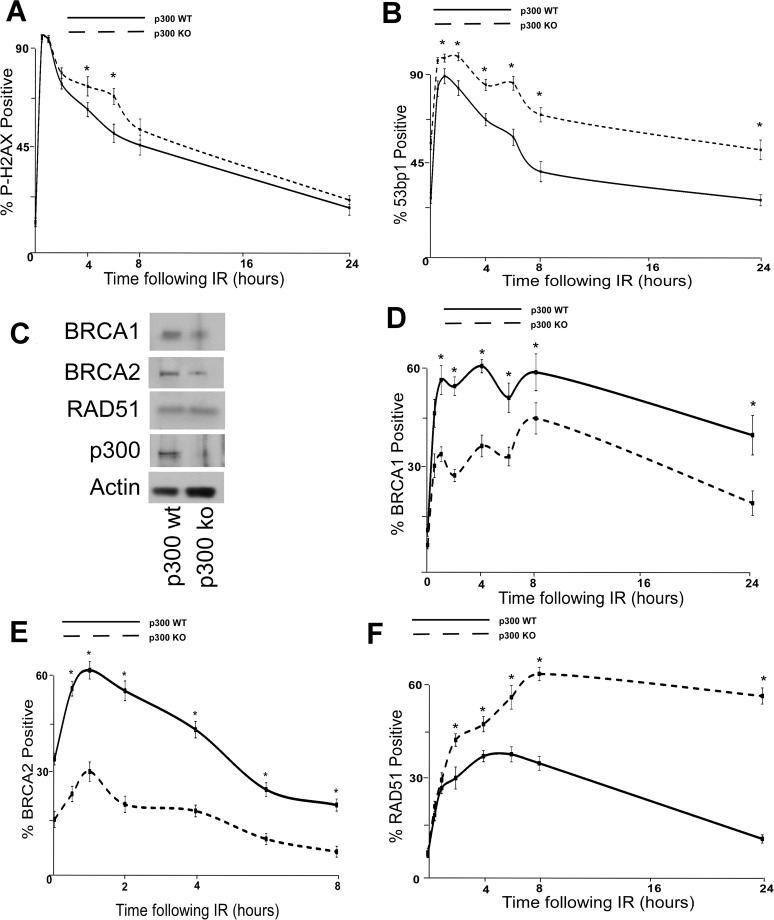
p300 is required for efficient double strand break repair. HCT 116 cells either with p300 knocked out (p300 ko) or with those p300ko cells complimented with wt p300 (p300 wt) were exposed to 4 gray of ionizing radiation and immunofluorescence microscopy was used to observe (A.) p-H2AX and (B.) 53bp1 foci formation for 24 hours after irradiation. (C.) Representative immunoblot of HCT 116 cells showing RAD51, BRCA2, BRCA1 and p300. Actin is included as a loading control. (D.)–(F.) HCT 116 were exposed to 4 gray of ionizing radiation and immunofluorescence microscopy was used to observe BRCA1, BRCA2 and RAD51 foci formation for 8 or 24 hours after irradiation. For all experiments, error bars depict standard errors of the mean. Similarly, * denote points where HCT 116 p300 ko cells significantly differed from control cells by student T-test with a p value ≤ 0.05. Finally, all experiments were conducted at least three times independently.

The dependence of β-HPV E6 mediated reduction of HDR on an intact p300 binding site suggests that p300 is required for optimal BRCA1/BRCA abundance and for proper formation/resolution of HDR repair foci. To formally test the role of p300 in DSB repair, the persistence of DSB markers (p-H2AX and 53bp1 foci) were measured in p300 knockout (p300 ko) and p300 complemented (p300 wt) HCT 116 cells [56]. Following IR, both of these markers are more persistent in HCT 116 p300 ko cells compared to HCT116 p300 wt cells (Fig. 4A-B and S14 Fig.). To determine if p300 is also required for typical HDR protein abundance, the amount of BRCA1, BRCA2 and RAD51 protein were determined by immunoblots of whole cell lysates from wt and p300 ko HCT 116 cells. Consistent with previous results (Fig. 2A-B), although there is no significant difference in the amount of RAD51 protein, there was less BRCA1 and BRCA2 protein in p300 ko HCT 116 cells compared to wt HCT 116 cells (Fig. 4C and S15 Fig.). IF microscopy was also used to determine whether p300 was required for typical BRCA1, BRCA2 and RAD51 foci formation and resolution in following IR. As predicted, p300 ko HCT 116 cells formed both BRCA1 and BRCA2 foci half as efficiently as p300 wt HCT 116 cells (Fig. 4D-E and S16 Fig.). Further, while RAD51 foci formation was not altered by the loss of p300, resolution of those foci was less efficient in p300 ko HCT 116 cells compared to p300 wt HCT 116 cells (Fig. 4F and S16 Fig.). As expected the repair deficiencies were more severe when the p300 gene was knocked out rather than present at low levels due to destabilization by HPV 5 and 8 E6.

**Fig 5:**
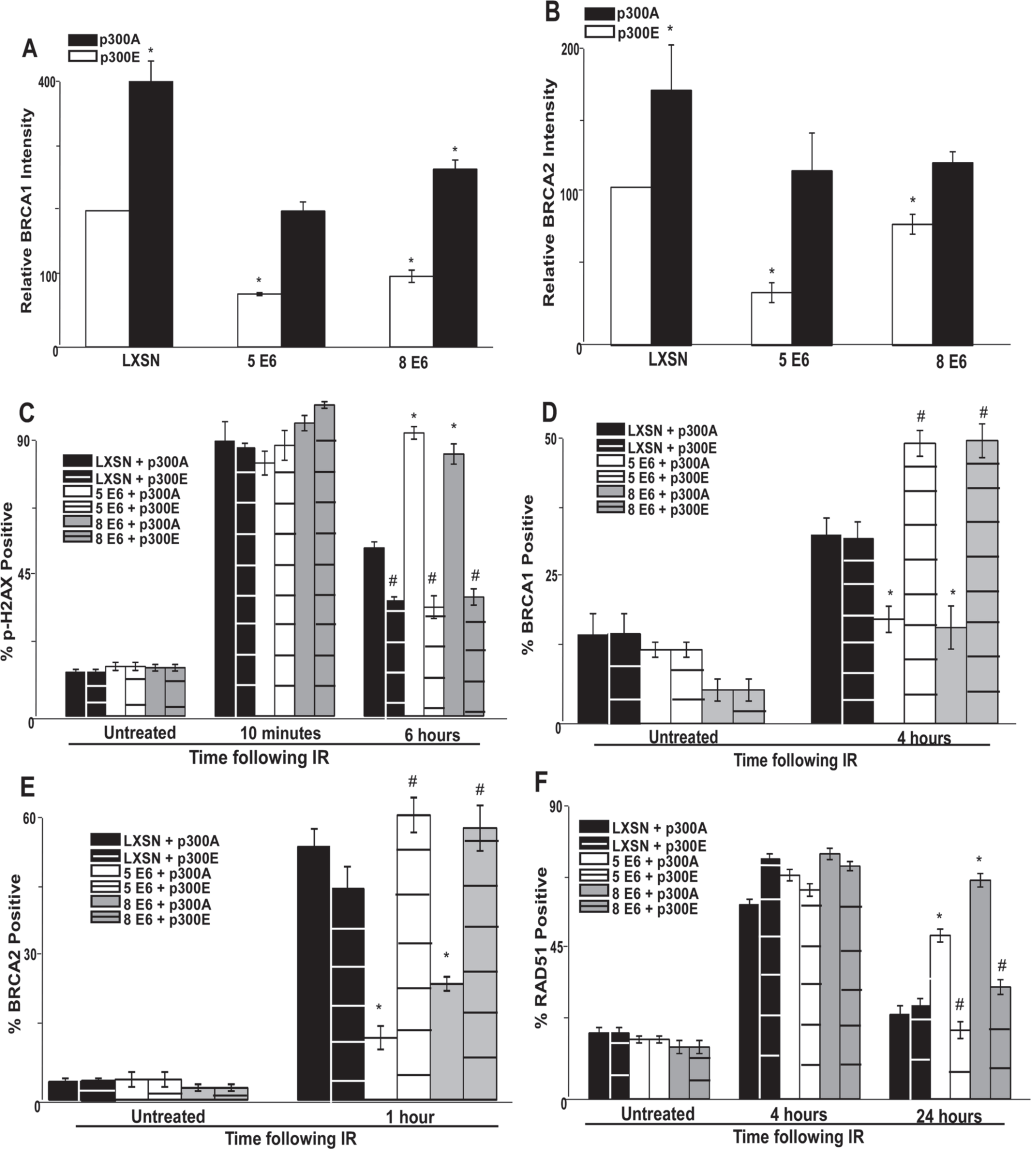
A mutant p300 that is resistant to β-HPV E6 mediated degradation rescues homology dependent repair of DSBs. Immunofluorescence microscopy was used to measure the intensity of (A.) BRCA1 and (B.) BRCA2 as well as foci positive cells for (C.) p-H2AX, (D.) BRCA1, (E.) BRCA2 and (F.) RAD51 foci persistence in HFK cells transfected with p300 expression vectors following exposure to 4 gray of ionizing radiation. These charts depict the percentage of foci positive cells at the indicated time points after irradiation. p300A denotes p300 S1834A mutant. p300E denotes p300 S1834E mutant. For all experiments, error bars depict standard errors of the mean. Similarly, * denote points where β-HPV E6 expressing cells significantly differed from the values of the corresponding control cells determined by student T-test and with a p value ≤ 0.05. # denotes a statistically significant difference within a cell line between cells transfected with p300A and p300E. Finally, all experiments were conducted at least three times independently.

To further tie degradation of p300 to the repair deficiencies observed in β-HPV 5 and 8 E6 expressing cells, the ability of a mutant p300 (p300 S1834E) that is resistant to β-HPV E6 mediated degradation to “rescue” DSB repair in β-HPV 5 and 8 E6 expressing cells was evaluated. Specifically, the effect of exogenous expression of this mutated p300 relative BRCA1 and BRCA2 levels were analyzed and found to be restored by expression of p300 S1834E. As a negative control, a previously described and inactive mutant p300, (p300 S1834A) was expressed in parallel in these cells [32,57]. Exogenous expression of p300 S1834A did not prevent β-HPV 5 and 8 E6 expression from increasing the persistence of p-H2AX foci seen 6 hours after IR treatment or from reducing BRCA1 and BRCA2 levels (Fig. 5A-C and S17 Fig.). In contrast, introduction of a p300 S1834E in β-HPV 5 or 8 E6 expressing cells restored DSB repair efficiency (Fig. 5C and S17 Fig.) [32,57].

The ability of exogenous p300 S1834E expression to rescue BRCA1 and BRCA2 foci formation following IR in β-HPV 5 and 8 E6 expressing HFKs was also examined. While BRCA1 and BRCA2 foci formation was diminished by β-HPV 5 and 8 E6 expression in the presence of p300 S1834A, the viral proteins failed to attenuate BRCA1 or BRCA2 foci formation when p300 S1834E was expressed (Fig. 5D-E and S18–S19 Fig.). Finally, the ability of β-HPV 5 and 8 E6 to increase the persistence of RAD51 foci following IR was mitigated by expression of p300 S1834E (Fig. 5F and S20 Fig.). β-HPV 5 and 8 E6 were still able to delay the resolution of RAD51 foci when p300 S1834A was expressed (Fig. 5F and S21 Fig.).

### β-HPV 5 and 8 E6 expression sensitizes cells to PARP inhibition and ionizing radiation

**Fig 6:**
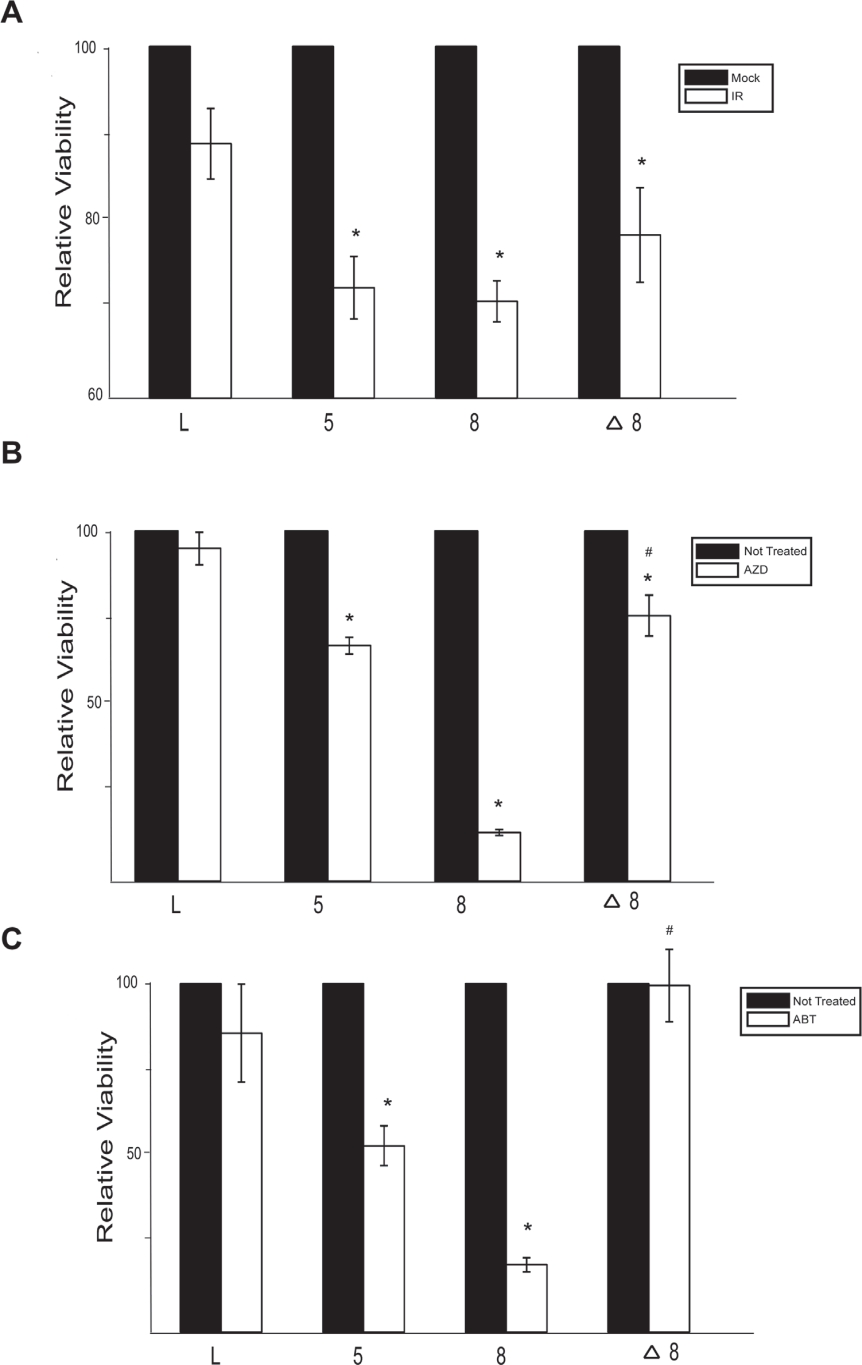
β-HPV 5 and 8 E6 increases sensitivity to ionizing radiation and PARP1 inhibition. (A.)–(C.) Relative viability of HFK cells exposed to either (A.) ionizing radiation (2 gray) or grown in media with PARP1 inhibitors (10 nM) (B.) AZD 2281 (AZD) and (C.) ABT 888 (ABT). n ≥ 3. Error bars represent standard errors of the mean. * denotes data that is statistically different from the similarly treated vector control as determined by student T-test with a p value ≤ 0.05. # denotes data that the value for HPV Δ8 E6 is statistically different from the similarly treated HPV 8 E6 cells as determined by student T-test with a p value ≤ 0.05. L denotes vector control (LXSN) HFKs. 5 denotes HPV 5 E6 expressing HFKs. 8 denotes HPV 8 E6 expressing HFKs. Δ8 denotes HPV Δ8 E6 expressing HFKs.

Because β-HPV 5 and 8 E6 inhibit DSB repair, cells expressing either of these viral proteins would likely be more sensitive to IR. To test this, the relative viability of control, β-HPV 5 and 8 E6 expressing HFKs was measured following IR. β-HPV 5 and 8 E6 expression significantly increased the sensitivity of HFKs to IR (Fig. 6A). Consistent with a role for p300 in the repair of DSBs, this sensitivity was less severe when β-HPV Δ8 E6 compared to β-HPV 8 E6 (Fig. 6A).

PARP1 plays a role in the repair of single strand breaks (SSBs) and through this repair prevents nicks from becoming DSBs [58]. Inhibition of PARP1 activity leads to an elevated number of DSBs and as a result loss of either BRCA1 or BRCA2 increases sensitivity to PARP1 inhibition [59]. To determine if expression of β-HPV 5 and 8 E6 expression will sensitize cells to PARP1 inhibition, cell viability was measured after growth in two PARP1 inhibitors, AZD-2281 and ABT-888. β-HPV 5 and 8 E6 expression sensitized cells to both AZD-2281 and ABT-888 (Fig. 6B-C). Notably, mutation of the p300 binding domain in β-HPV 8 E6 reduced the viral proteins ability to increase the toxicity of PARP1 inhibition (Fig. 6B-C)

## Discussion

β-HPV infections may be involved in the development of some skin cancers [4,5,7–9]. Since β-HPV genomes are present only in very low copy numbers in tumors, they are hypothesized to contribute to oncogenesis indirectly by increasing the mutagenic potential of UV exposure. In support of this theory, we have previously shown that by attenuating ATR expression in a p300 dependent manner, β-HPV 5 and 8 E6 disrupt UV damage repair making it significantly more likely that the DNA crosslinks caused by UV exposure become more deleterious DSBs [29]. Here, we show for the first time that β-HPV 5 and 8 E6 disrupt repair of these DSBs induced by IR through the reduced expression and repair foci formation of two central proteins involved in the HDR of the DSBs, BRCA1 and BRCA2 (Fig. 1 and 2A-D). Additionally, we show that while neither resection nor RAD51/RPA70 exchange is perturbed, RAD51 foci resolution is delayed by β-HPV 5 and 8 E6 (Fig. 2E-F). Using a GFP based assay, we show that these viral proteins significantly decreased the efficiency of HDR (Fig. 3). Mechanistically, these phenotypes require an intact p300 binding domain in β-HPV 8 E6, suggesting that the viral proteins abrogate HDR by destabilizing p300 (Figs. 1–3). Supporting this mechanism, targeted knockout of the p300 gene displayed strikingly similar defects in HDR, including attenuated expression and foci formation of both BRCA1 and BRCA2 as well as increased persistence of RAD51 foci and DSB makers (Fig. 4). Moreover, the ability of β-HPV 5 and 8 E6 to attenuate DSB repair could be blocked by exogenous expression of a mutant p300 (p300 S1834E) that is resistant to destabilization by the viral proteins (Fig. 5). Finally, β-HPV 5 and 8 E6 expression sensitizes cells to both PARP1 inhibition and IR exposure, but the increased toxicity is not seen in β-HPV Δ8 E6 expressing cells (Fig. 6). When combined with previous reports [29,31] that β-HPV 5 and 8 E6 expression increases UVB-induced DSBs, this paper provides a plausible mechanism for the role of β-HPV infections as co-factors in skin cancer development and substantially increases the understanding of HDR.

### β-HPV 5 and 8 E6 proteins target p300 to alter multiple cellular pathways

Our group and others have shown that certain β-HPV E6 proteins (namely HPV 5, 8 and to a lesser extent 38 E6) manipulate their host cell similarly to the way HR α-HPV E6 (HR-α HPV E6) proteins alter their host cell [16,18,23,28,29,60–66]. Some of these changes occur through mechanisms common to both HR-α and β-HPV E6 proteins, like the modulation of apoptosis by BAK degradation [64,65]. In other cases, similar alterations are achieved through diverse mechanisms. Specifically, while β-HPV 5 and 8 E6 are not capable of degrading p53, they attenuate p53 signaling in response to UVB exposure by reducing the expression and activation of ATR, thus delaying p53 stabilization [19,20,29,61,62].

HR α-HPV E6’s degradation of p53 allows the virus to alter its host cell’s response to diverse cellular stimuli that would otherwise lead to cell cycle arrest, apoptosis and/or senescence. This strategy of targeting a center spoke of multiple pathways allows HR α-HPVs to efficiently reprogram their host cell, despite only expressing 8 proteins. The data presented here demonstrates that destabilization of p300 allows β-HPVs to modulate DSB repair (Figs. 1–3). Further, we have previously demonstrated that β-HPV E6-mediated decrease in p300 alters the cell’s response to differentiation, UVB-induced DNA damage and erroneous mitosis [29,30,32]. Together, our work establishes that some β-HPVs target p300 for destabilization to efficiently reprogram their host cell much like HR-α HPVs promote p53 degradation to alter the host cell.

Interestingly, deletion of the p300 binding domain from HPV 8 E6 does not completely restore some aspects of HDR. Specifically, BRCA1 foci positive cells are reduced in keratinocytes expressing HPV Δ8 E6 compared to control cells. Similarly, the kinetics of RAD51 resolution are not fully restored by the mutant. This suggests HPV 8 E6 can attenuate HDR through both p300 dependent and independent mechanisms. The ability to target the same pathway through multiple mechanisms has been reported for other papillomavirus proteins and often signifies a phenotype that is quintessential to the viral life cycle, suggesting that inhibition of HDR provides a sizable selective advantage to β-HPV 8 [67,68].

The p300 binding domain of β-HPV 5/8 E6 may also contribute to reduced BRCA1/2 protein levels independent of its effects on transcription, as the reduction in mRNA levels is less dramatic than the reduction in protein levels. One possibility for this observation is that the same sequences of HPV 5/8 E6 responsible for p300 binding also reduce the stability of BRCA1/2 protein. Alternatively, there may not be a one to one correlation of mRNA transcripts to translated protein making it possible for a 50% reduction in mRNA to result in a 70% reduction in protein.

### Robust BRCA1/2 activitation is not required for RPA or RAD51 foci formation

In the classical model of HDR of DSBs, BRCA1 acts at two points in the pathway [36,38]. First, it promotes resection of DNA near the lesion [41] and in combination with BRCA2 and PALB2, it promotes RPA:RAD51 exchange on the ssDNA [45,46]. Here, we show that not only is p300 required for robust BRCA1 as well as BRCA2 expression (Fig. 2) [39,40]. Further, we demonstrate that BRCA1 and BRCA2 foci formation as well as resection (as measured by RPA foci formation) and RPA:RAD51 exchange can occur without optimal BRCA1/2 focus formation (Figs. 2 and 4). These observations are not necessarily at conflict with the well characterized process of HDR. Instead, our data suggests that the amount of BRCA1 activation that is typically seen in response to IR induced DSB is in excess of the amount required for resection (Fig. 2 and 4). Although the kinetics of RPA:RAD51 exchange are not altered by lack of optimal BRCA1 and BRCA2 response, RAD51 may be loaded at high enough levels to detect by immunofluorescence microscopy but not at too low an abundance to facilitate strand exchange or efficient HDR. Alternatively, there may be additional defects in RAD51 unloading that explain the increased RAD51 persistence. Notably, the ability to form RAD51 foci in the absence of BRCA1 activation has been reported in cells lacking the ability to complete NHEJ [69–71]. As a result, the formation of RAD51 foci in β-HPV 5 and 8 E6 expressing cells indicates that these viral proteins may disrupt NHEJ as well as HDR. Further supporting this idea, HPV 38 E6 expression is capable of attenuating HDR, but does not increase the persistence of DSBs suggesting that this viral protein may be able to partially inhibit HDR but not NHEJ.

### β-HPV 5 and 8 E6 expression substantially increases the mutagenic potential of UVB exposure

We present multiple lines of evidence demonstrating that β-HPV 5 and 8 E6 disrupt HDR (Figs. 1–3). Not only is this a substantial advancement in the knowledge of the reprograming of host cells by β-HPV proteins, but also a marked increase in the understanding of how deleterious β-HPV infections can be. While the ability of β-HPV E6 expression to attenuate thymine dimer repair provided a plausible mechanism for β-HPV infections to act as a co-factor along with UV, misrepair of thymine dimer results in a minimal loss of genomic information [29,31]. Although the lack of a proper G1 arrest in cells expressing β-HPV 5 and 8 E6 increases the likelihood that a thymine dimer becomes a DSB, DSBs are often repaired in an error-free manner via HDR. Here, we show that this is significantly less likely to occur in cells that have been infected with β-HPV 5 or 8. Instead, if a β-HPV infected cell is damaged by UV light not only will the resulting crosslinked DNA be more likely to become a DSB, but the cell will also have difficulty repairing the DSB. Misrepair of the DSB can result in deletions and chromosomal rearrangements. Ultimately, we add to the already strong argument that β-HPV infections abrogate proper repair of UVB-damaged DNA making UV exposure more mutagenic and providing a mechanism to explain the proposed role of β-HPV infections as a co-factor for skin cancers.

## Materials and Methods

### Chromatin immunoprecipitation

Chromatin immunoprecipitations were performed using the Enzymatic Chromatin IP (Magnetic bead) kit (Cell Signaling Technology), as per the manufacturer's instructions, with minor modifications. Briefly, chromatin from fixed cells was digested to a size range of 150–1000 bases with micrococcal nuclease, followed by brief sonication to disrupt the nuclear membrane. Solubilized chromatin was immunoprecipitated with antibodies to p300(N-15) or IgG control. Antibody-chromatin complexes were pulled-down using ChIP-grade protein-G magnetic beads, washed and then eluted. After cross-link reversal and proteinase K treatment, immunoprecipitated DNA was extracted with phenol-chloroform, and ethanol precipitated. Real-time RT-PCR was performed using SYBR green and primers to the BRCA1, BRCA2 and GAPDH promoters. Standard curves were calculated using serial dilutions of the input sample, and used to calculate the relative amount of product amplified in each reaction. Results were calculated based on the relative enrichment of protein over that seen at the GAPDH promoter and then normalized to LXSN (vector) control.

Primers used for ChIP are as follows:

GAPDH: Forward. 5’-atgctgagtgtacaagcgttttct-3’ Reverse. 5’-cactatgccaccccaggaat-3’

BRCA2: Forward. 5’-caagcagatgatgtttcctgtcc-3’ Reverse. 5’-agaactaagggtgggtggtgtagc-3’

BRCA1: Forward. 5’-tttcgtattctgagaggctgctg-3’ Reverse. 5’-atttatctgtaattcccgcgctt-3’

### Immunofluorescence microscopy

HFKs (control, HPV 5 E6, 8 E6, Δ8E6, 38 E6 and 16 E6) were seeded on cover slips or in Greiner glass bottom 96 well dishes and grown overnight. Following exposure to IR (4 gy), cells were washed and fixed with 4% paraformaldehyde at the indicated times. Then, the cells were permeabilized with .5% Triton-X-PBS. The cells were then blocked with Blocking Buffer (3% BSA, .1% Tween20, PBS). The cover slips and 96 well dishes were stained with the following antibodies: BRCA1, BRCA2, p-H2AX, 53bp1, RAD51, RPA70, p300. The primary antibodies were added in Blocking Buffer and incubated overnight. The next day, the cells were stained with appropriate Alexa Fluor secondary antibodies from and Hoechst 33342 for one hour.

### Immunofluorescence microscopy

Images of stained cells were acquired using a Cellomic ArrayScan VTI using a 20 x 0.40 NA objective. Image analysis was performed using Cellomics ArrayScan HCS Reader. Nuclear staining was used to delineate cells. After background correction, the quantity of nuclear foci was measured. Approximately 400 cells were analyzed in each well. Each time point and cell line was repeated in triplicate. For BRCA1 and BRCA2, cells were considered positive if they had greater than 25 foci. For RAD51 and RPA70, cells were considered positive if they had greater than 10 foci per cell.

### Immunoblotting

Whole cell extracts were prepared by mechanically detaching cells and resuspending in WE16th lysis buffer (previously described [32]). Lysates were then sonicated and clarified by centrifugation. Equal amounts of protein lysates (15–30 μg) were electrophoresed on SDS-polyacrylamide gels and transferred to Immobilon-P membranes. These membranes were then exposed to primary antibodies against BRCA1, BRCA2, RAD51, RPA70, p300, Nucleolin and Actin. After exposure to the corresponding HRP-conjugated secondary antibody, proteins were visualized using HRP substrate. For quantification, densitometry was done on scanned unsaturated images using the ImageJ software.

### Tissue culture

U2OS cells are derived from an osteosarcoma. The integration of a single copy DR-GFP construct into these cells is described in [50]. Primary human foreskin keratinocytes (HFKs) were derived from neonatal human foreskins and grown in EpiLife medium supplemented with calcium chloride (60 μM), Human keratinocyte growth supplement and penicillin-streptomycin. Multiple derivations of HFKs from neonatal human foreskins were utilized in this work. Following viral transduction and selection, HPV E6 expression was confirmed using rtPCR and by immunoblots to confirm the expected reduction of p300 [32].

All transient transfections of HFKs were done using TransIT Keratinocyte transfection reagent and assessed 48 hours later. All transient transfections of U2OS cells were done with Xtreme Gene according to the manufacturer’s instructions.

P300 ko and p300 wt HCT 116 cells were generous gifts from Dr. Carlos Caldas at the Cancer Research UK Cambridge Institute. The generation of these cells has been described previously [56], but briefly involved the targeted knock out of the second exon of p300 (p300 ko cells) and followed by restoration of p300 expression by transfection of a p300 expression vector (p300 wt cells).

### DR-GFP assay

The development of this assay, along with the basic protocol is described here [50]. Briefly, relative homology-dependent repair of I-SceI generated DSBs was measured 24 hours after transfection of an I-SceI expression vector by analyzing GFP positive cells using a BD Canto 1 instrument and quantified using FloJo.

### PARP1 inhibition

Both inhibitors were dissolved in DMSO. ABT 888 and AZD 2281 were used at a concentration of 10 nM.

### RT-PCR

RNA was isolated with Trizol reagent. 1 μg of total RNA was reverse transcribed to generated cDNA using the iScript cDNA synthesis kit. As a negative control parallel samples were run without reverse transcriptase. Non-quantitative PCR amplification was then performed to identify 100 bp amplicons with E6 and 36B4 primers as previously described [64]. For real-time RT-PCR, RNA was isolated and reverse transcribed as above and quantitative real-time PCR was performed using an ABI 9700 sequence detection system (Applied Biosystems, Foster City, CA). Amplification was carried out using TaqMan primer/probes: HPRT (Hs01003267_m1), GAPDH (Hs02758991_m1), BRCA1 (Hs01556193_m1 and Hs01556189_m1) and BRCA2 (Hs00609073_m1 and H201037421_m1) according to the manufacturer's instructions (Applied Biosystems, Foster City, CA). Reactions were performed in triplicate in a 25 μL volume, with the following cycle parameters: enzyme activation (10 minutes at 95°C), followed by 40 cycles (each cycle consisting of 15 seconds at 95°C and 1 min at 60°C). Data analysis was performed using the comparative threshold cycle method (Applied Biosystems, Foster City, CA) to determine expression levels.

### Viability assay

This assay has been described in detail previously [29]. Briefly, 12 hours after seeding, 10000 cells were either exposed to ionizing radiation or switched to media containing PARP1 inhibitors. After three days, cells were stained with crystal violet for one hour. The plates were then washed and dried. Crystal violet stain was then eluted from the cells in 10% acetic acid and the stain was quantitated by measuring absorbance at 590 nanometers using KC Junior software and a μQuant spectrophotometer.

### UVB irradiation

Cells were seeded and grown overnight. The following morning, cells were washed once with PBS and then irradiated through a thin film of PBS with the amount of UVB irradiation indicated in the figure. Fresh media was then added to the cells. The UVB source is a parallel bank of two FS20t12/UVB bulbs (Solarc Systems, Inc., Barrie, ON, Canada) with an output range of 280 to 320 nm. The UVB output is measured with an IL1400A radiometer coupled with the SEL240/UVB-1/TD UVB detector (International Light, Peabody, MA).

### Chromosome fragility assay

75,000 U2OS cells were seeded per well of a 6 well plate. 12 hours later, 0, 2.5 or 5 ng/ml of Mitomycin C was added to the media. Following 48–72 hours growth in Mitomycin C media, cells were collected by trypsinization. Cells were then pelleted and the supernatant was removed. Cells were resuspended in 0.051 M KCL and incubated at 37C for 25 minutes. Cells were again pelleted, supernatant was removed, and cells were resuspended in fixative solution (4 parts methanol to 1 part acetic acid). After 2 washes in fixative solution, cells were resuspended in approximately half an ml of fixative solution before being plated onto slides. Once plated cells were stained with Giemsa. Images of mitotic chromosomes were taken using an Evos FL Auto microscope (Life Technologies). At least 500 chromosomes were accessed for breaks in triplicate for each sample.

## Supporting Information

S1 Fig Confirmation of HPV E6 expression. Semi-quantitative rt-PCR on HPV expressing HFK cells was performed with primers specific to each HPV E6. As a loading control, 36b4 was also amplified.
(TIF)

S2 Fig BRCA1 foci formation in response to UV. Immunofluorescence microscopy was used to measure BRCA1 foci in HFK cells following exposure to 10 mJ/cm^2^UVB. (A.) This chart depicts the percentage of BRCA1 foci positive cells after UVB exposure (B.) This chart depicts the percentage of BRCA1 foci positive cells after UVB exposure corrected for the reported frequency of UVB-induced DSBs. For both charts, x-axis represents time (in hours) after UVB exposure. Error bars depict the standard error of the means. n>5 at all points.
(TIF)

S3 Fig β-HPV 8 E7 does not prevent β-HPV 8 E6 from attenuating DSB repair. Immunofluorescence microscopy was used to measure p-H2AX foci in control cells (black) as well as cells expressing HPV 8 E6 alone (blue) or in combination with HPV 8 E7 (blue stripes) following exposure to 4 gray of ionizing radiation. Error bars depict the standard error of the means. n = 3 for all data points.
(TIF)

S4 Fig β-HPV 5 and 8 E6 reduce transcription of BRCA1 and BRCA2. A. BRCA1 mRNA levels measured by q-rtPCR and first normalized to the housekeeping gene HPRT or GAPDH mRNA levels before being set relative to LXSN. B. Enrichment of p300 at the IgG or BRCA1 promoter. C. BRCA2 mRNA levels measured by q-rtPCR and first normalized to the housekeeping gene HPRT or GAPDH mRNA levels before being set relative to LXSN. D. Enrichment of p300 at the IgG or BRCA2 promoter.
(TIF)

S5 Fig β-HPV 38 E6 does not attenuate BRCA1 or BRCA2 expression. Representative immunoblot of HFK cells showing BRCA2 and BRCA1. Nucleolin is included as a loading control.
(TIF)

S6 Fig β-HPV 5 and 8 E6 attenuate BRCA1 foci formation in response to IR. Representative images of cells 4 hours after exposure to 4 gray of IR with both BRCA1 (pink) and nuclei (blue) staining.
(TIF)

S7 Fig β-HPV 5 and 8 E6 reduce the number of BRCA1 and BRCA2 foci per cell. These charts depict the average number of (A.) BRCA1 and (B.) BRCA2 foci per cell following IR exposure. Error bars depict the standard error of the means. n>5 at all points.
(TIF)

S8 Fig BRCA2 foci formation following IR is diminished by β-HPV 5 and 8 E6. Representative images of cells 90 minutes after exposure to 4 gray of IR with both BRCA2 (pink) and nuclei (blue) staining.
(TIF)

S9 Fig RPA foci formation and resolution is unchanged by β-HPV 5 and 8 E6. Representative images of cells 90 minutes after exposure to 4 gray of IR with both RPA (pink) and nuclei (blue) staining.
(TIF)

S10 Fig Resolution of RAD51 foci is delayed by β-HPV 5 and 8 E6. Representative images of cells 24 hours after exposure to 4 gray of IR with both RAD51 (pink) and nuclei (blue) staining.
(TIF)

S11 Fig β-HPV 5 and 8 E6 attenuate DSB foci resolution in U20S cells. Representative images of cells 6 hours after exposure to 4 gray of IR with both p-H2AX (pink) and nuclei (blue) stained.
(TIF)

S12 Fig HPV 5 and 8 E6 attenuate BRCA1 and BRCA2 levels in U2OS cells. Densitometry of immunoblots of BRCA1 (blue) and BRCA2 (red). The amount of protein in each sample was normalized to the corresponding amount of nucleolin and then data was set relative to vector control (LXSN) cells.
(TIF)

S13 Fig HPV 5 and 8 E6 attenuate BRCA1 and BRCA2 foci formation in U2OS cells. U2OS cells expressing either control, HPV 5 E6, HPV 8 E6 or HPV Δ8 E6 were exposed to 4 gray of ionizing radiation and immunofluorescence microscopy was used to observe (A.) BRCA1 and (B.) BRCA2 foci formation 4 hours after irradiation.
(TIF)

S14 Fig HPV 38 E6 expression reduces the frequency of HDR in U2OS cells. Representative samples of FACS profiles used to generate data shown in Fig. 3C.
(TIF)

S15 Fig p300 is required for efficient DSB repair. Representative images of cells following exposure to 4 gray of IR with either p-H2AX (pink) or 53bp1 (pink) as indicated and nuclei (blue) stained.
(TIF)

S16 Fig p300 is required for robust expression of BRCA1 and BRCA2. A. Densitometry of immunoblots of BRCA1 and BRCA2 in HCT cells with (black) and without p300 (white). The amount of protein in each sample was normalized to the corresponding amount of nucleolin and then data was set relative to wild type p300 HCT cells.
(TIF)

S17 Fig p300 is required for robust HR repair foci formation and resolution following IR. Representative images of cells after exposure to 4 gray of IR with either A. BRCA1 (pink) and nuclei (blue) stained, (B.) BRCA2 (pink) and nuclei (blue) stained, or (C.) RAD51 (pink) and nuclei (blue) stained.
(TIF)

S18 Fig p300E rescues p-H2AX foci resolution in β-HPV 5 and 8 E6 expressing cells. Control, 5 E6, or 8 E6 expressing cells transfected with p300A or p300E as indicated before being stained for p-H2AX (pink) and nuclei (blue) cells 0 minutes, 10 minutes or 6 hours after exposure to 4 gray of IR.
(TIF)

S19 Fig p300E rescues BRCA1 foci formation in β-HPV 5 and 8 E6 expressing cells. Control, 5 E6, or 8 E6 expressing cells transfected with p300A or p300E as indicated before being stained for BRCA1 (pink) and nuclei (blue) cells 4 hours after exposure to 4 gray of IR.
(TIF)

S20 Fig p300E rescues BRCA2 foci formation in β-HPV 5 and 8 E6 expressing cells. Control, 5 E6, or 8 E6 expressing cells transfected with p300A or p300E as indicated before being stained for BRCA2 (pink) and nuclei (blue) cells 1 hour after exposure to 4 gray of IR.
(TIF)

S21 Fig p300E rescues RAD51 foci resolution in β-HPV 5 and 8 E6 expressing cells. Control, 5 E6, or 8 E6 expressing cells transfected with p300A or p300E as indicated before being stained for RAD51 (pink) and nuclei (blue) cells 0 minutes, 4 or 24 hours after exposure to 4 gray of IR.
(TIF)
