# Supplementary figures and images for: β-HPV 5 and 8 E6 Disrupt Homology Dependent Double Strand Break Repair by Attenuating BRCA1 and BRCA2 Expression and Foci Formation
Source: PLoS Pathog. 2015 Mar 24;11(3):e1004687. doi: 10.1371/journal.ppat.1004687 (PMC4372404; doi:10.1371/journal.ppat.1004687)

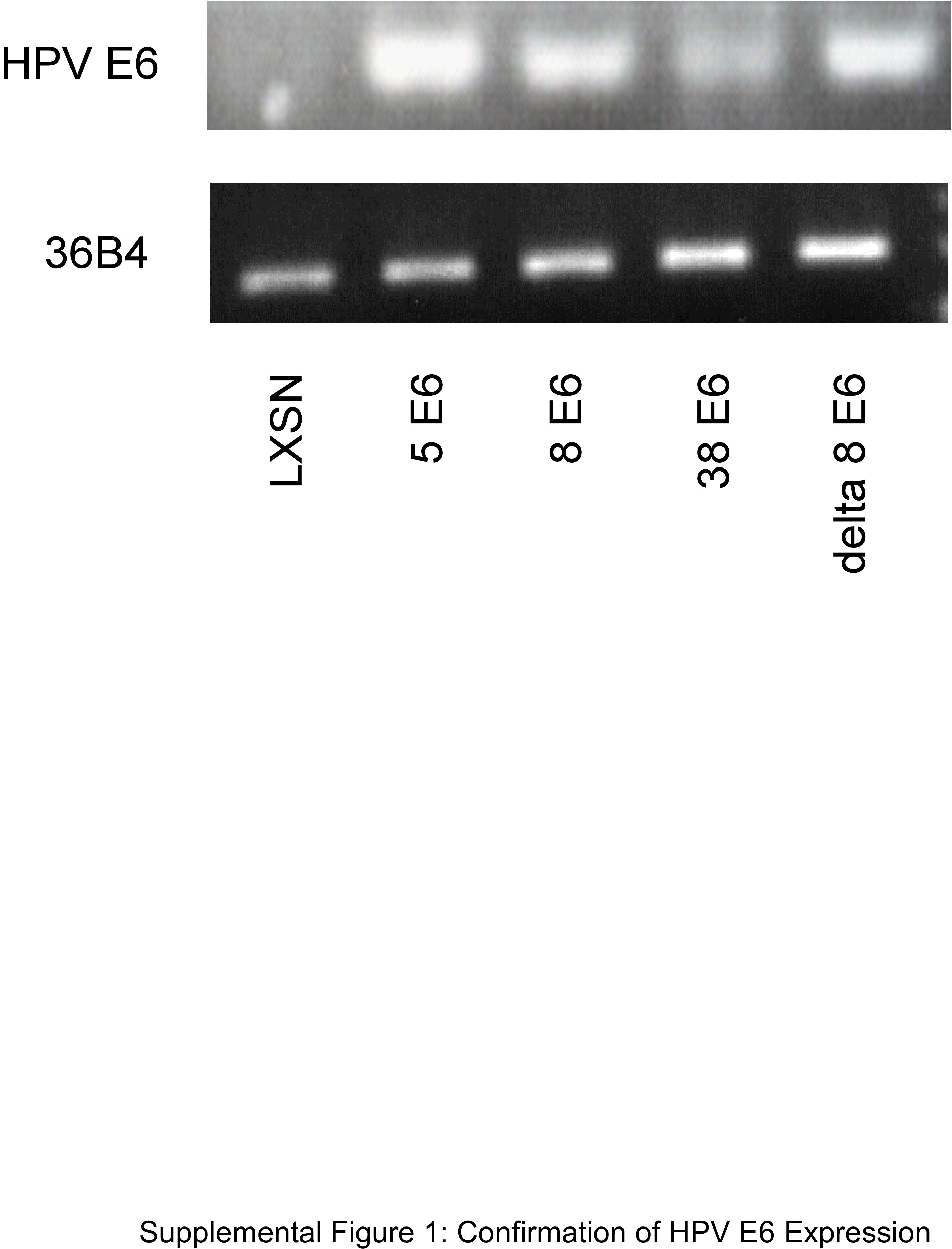

Supplement: S1 Fig — Semi-quantitative rt-PCR on HPV expressing HFK cells was performed with primers specific to each HPV E6. As a loading control, 36b4 was also amplified. (TIF) [file ppat.1004687.s001.tif]

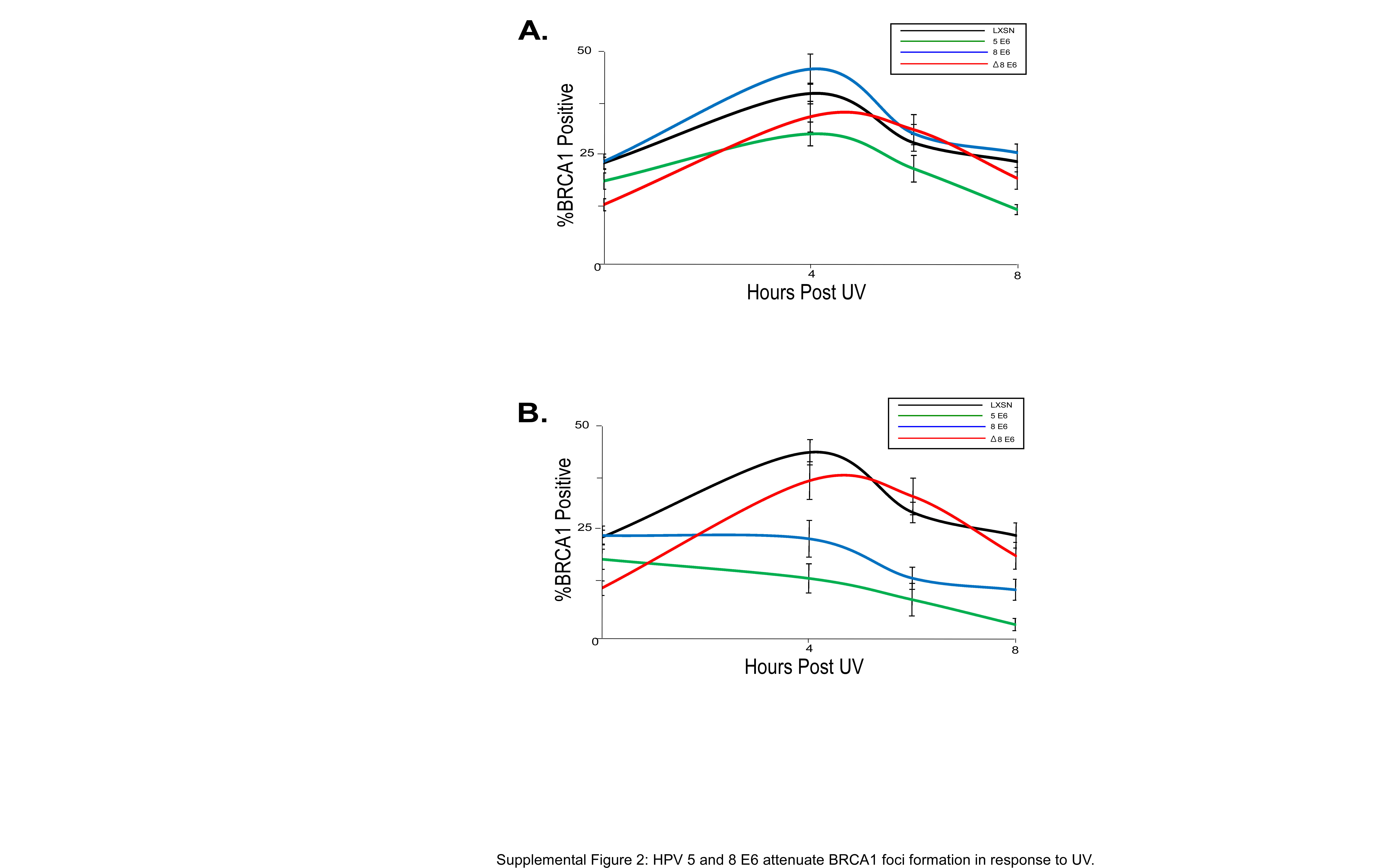

Supplement: S2 Fig — Immunofluorescence microscopy was used to measure BRCA1 foci in HFK cells following exposure to 10 mJ/cm2UVB. (A.) This chart depicts the percentage of BRCA1 foci positive cells after UVB exposure (B.) This chart depicts the percentage of BRCA1 foci positive cells after UVB exposure corrected for the reported frequency of UVB-induced DSBs. For both charts, x-axis represents time (in hours) after UVB exposure. Error bars depict the standard error of the means. n>5 at all points. (TIF) [file ppat.1004687.s002.tif]

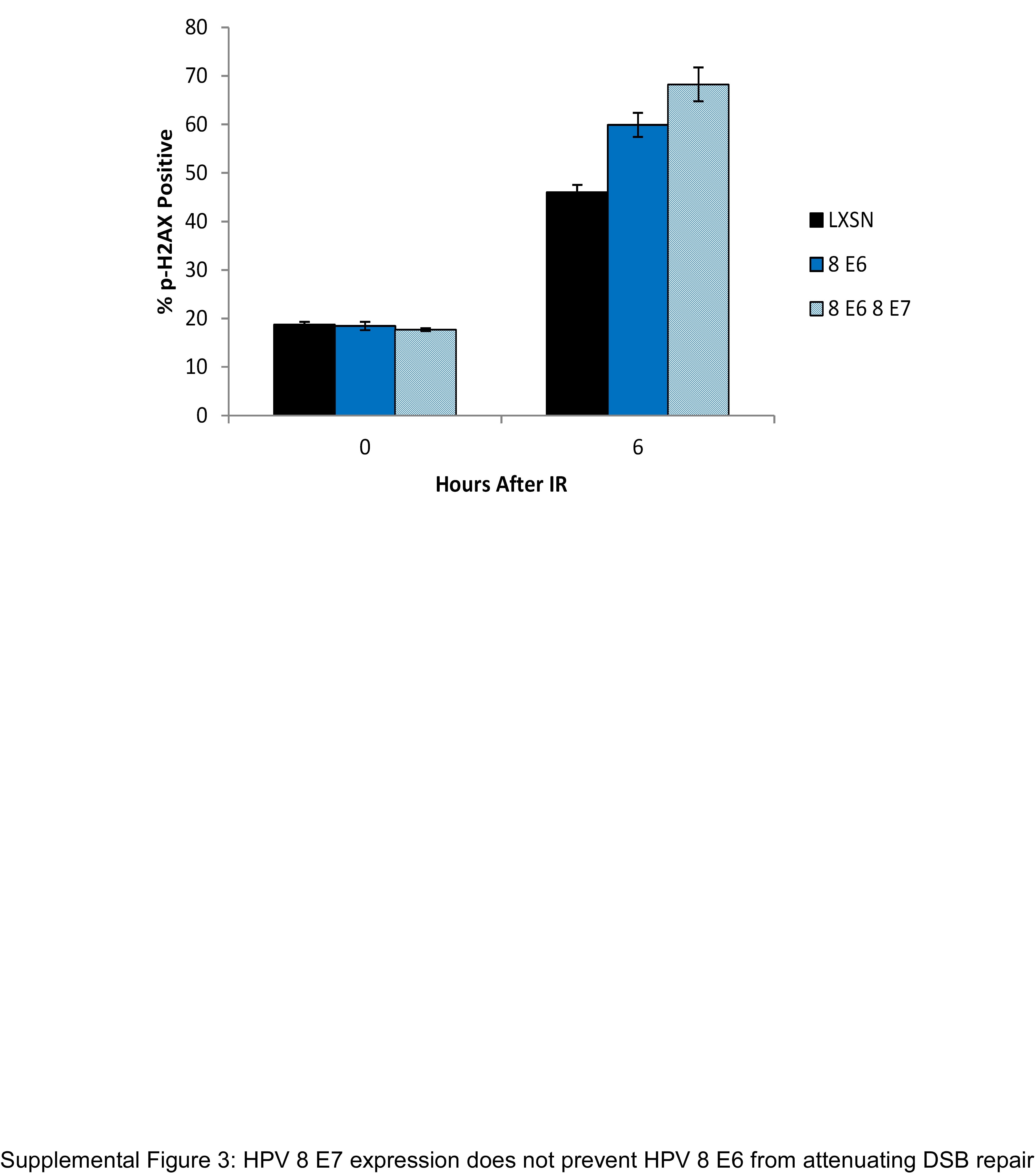

Supplement: S3 Fig — Immunofluorescence microscopy was used to measure p-H2AX foci in control cells (black) as well as cells expressing HPV 8 E6 alone (blue) or in combination with HPV 8 E7 (blue stripes) following exposure to 4 gray of ionizing radiation. Error bars depict the standard error of the means. n = 3 for all data points. (TIF) [file ppat.1004687.s003.tif]

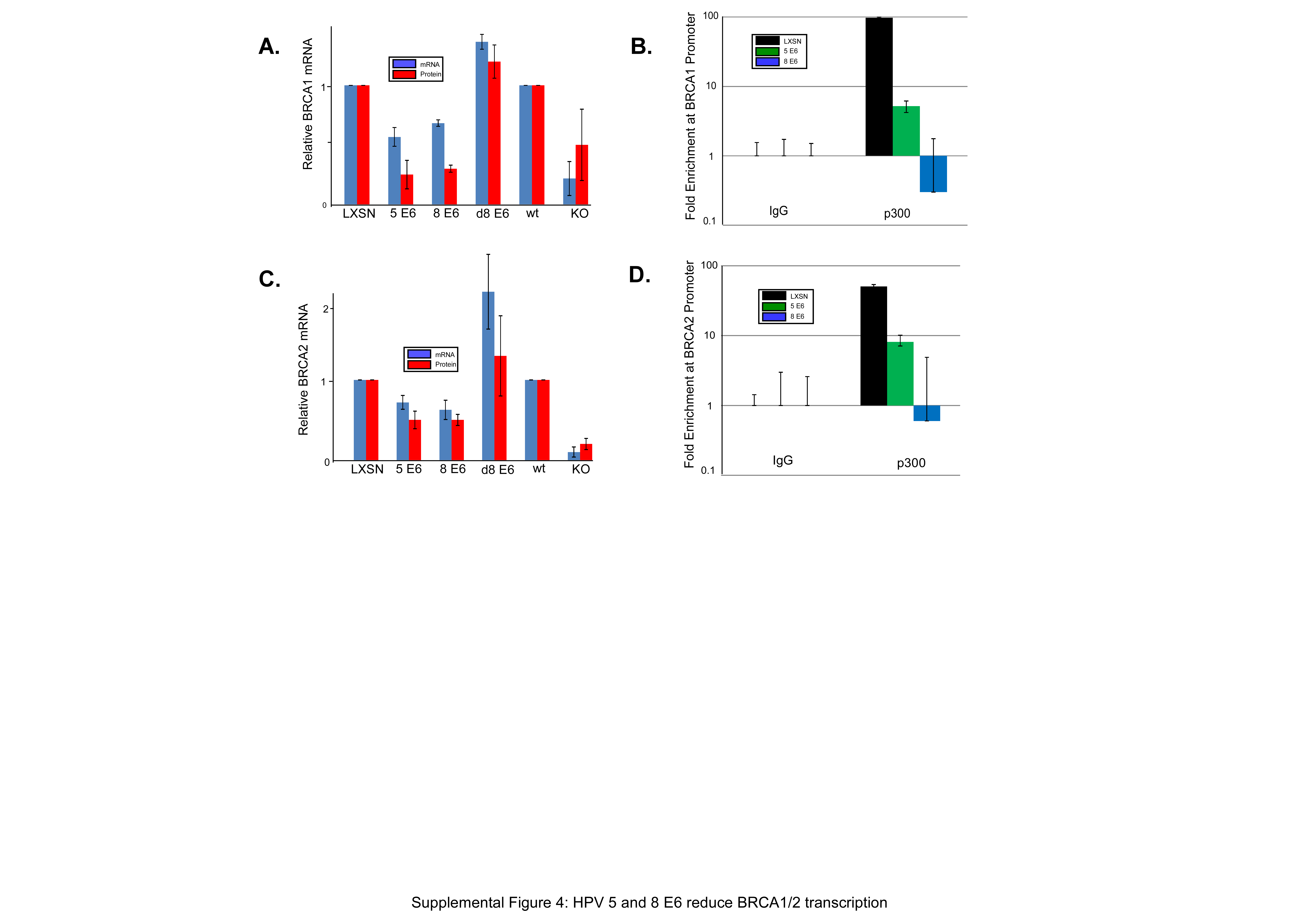

Supplement: S4 Fig — A. BRCA1 mRNA levels measured by q-rtPCR and first normalized to the housekeeping gene HPRT or GAPDH mRNA levels before being set relative to LXSN. B. Enrichment of p300 at the IgG or BRCA1 promoter. C. BRCA2 mRNA levels measured by q-rtPCR and first normalized to the housekeeping gene HPRT or GAPDH mRNA levels before being set relative to LXSN. D. Enrichment of p300 at the IgG or BRCA2 promoter. (TIF) [file ppat.1004687.s004.tif]

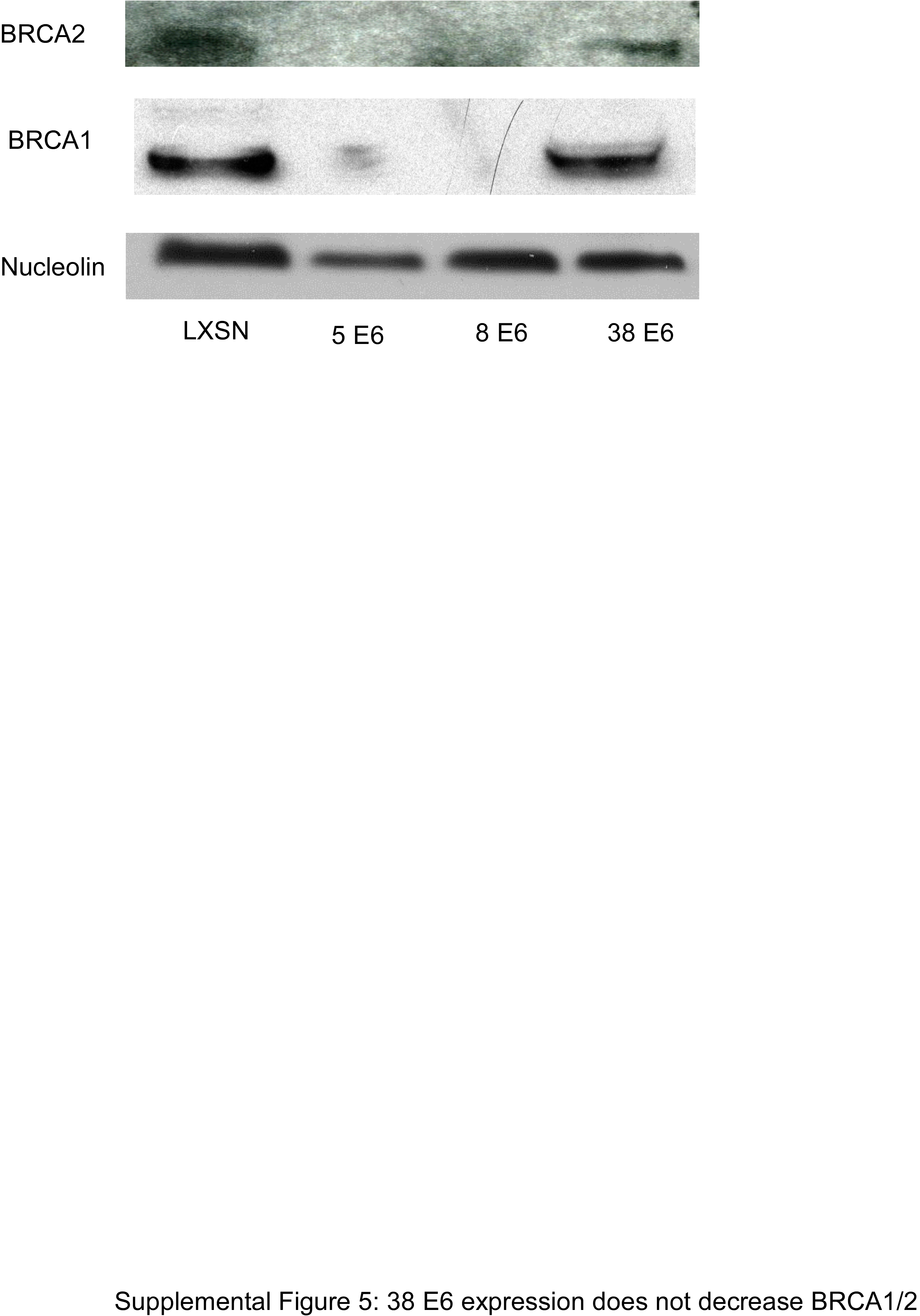

Supplement: S5 Fig — Representative immunoblot of HFK cells showing BRCA2 and BRCA1. Nucleolin is included as a loading control. (TIF) [file ppat.1004687.s005.tif]

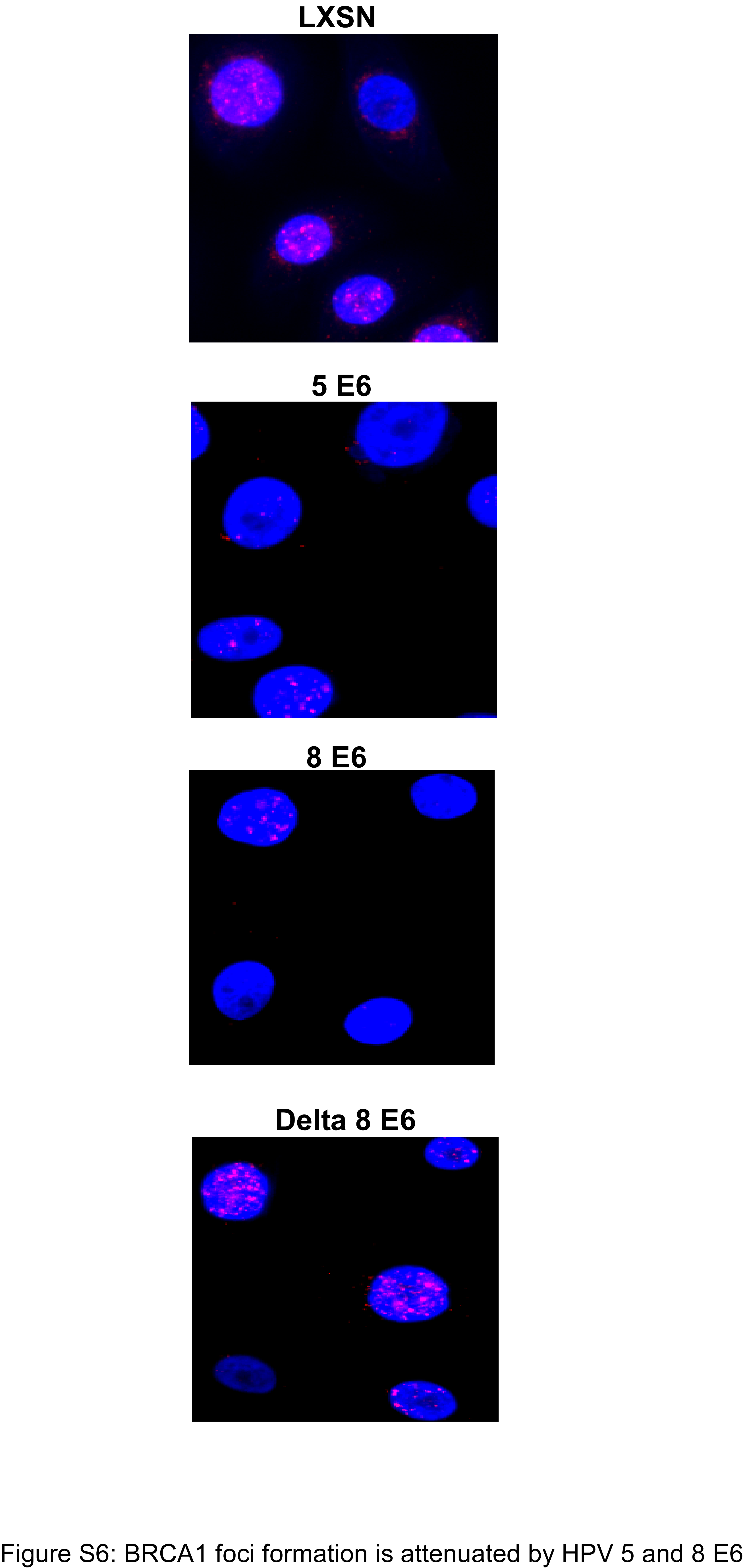

Supplement: S6 Fig — Representative images of cells 4 hours after exposure to 4 gray of IR with both BRCA1 (pink) and nuclei (blue) staining. (TIF) [file ppat.1004687.s006.tif]

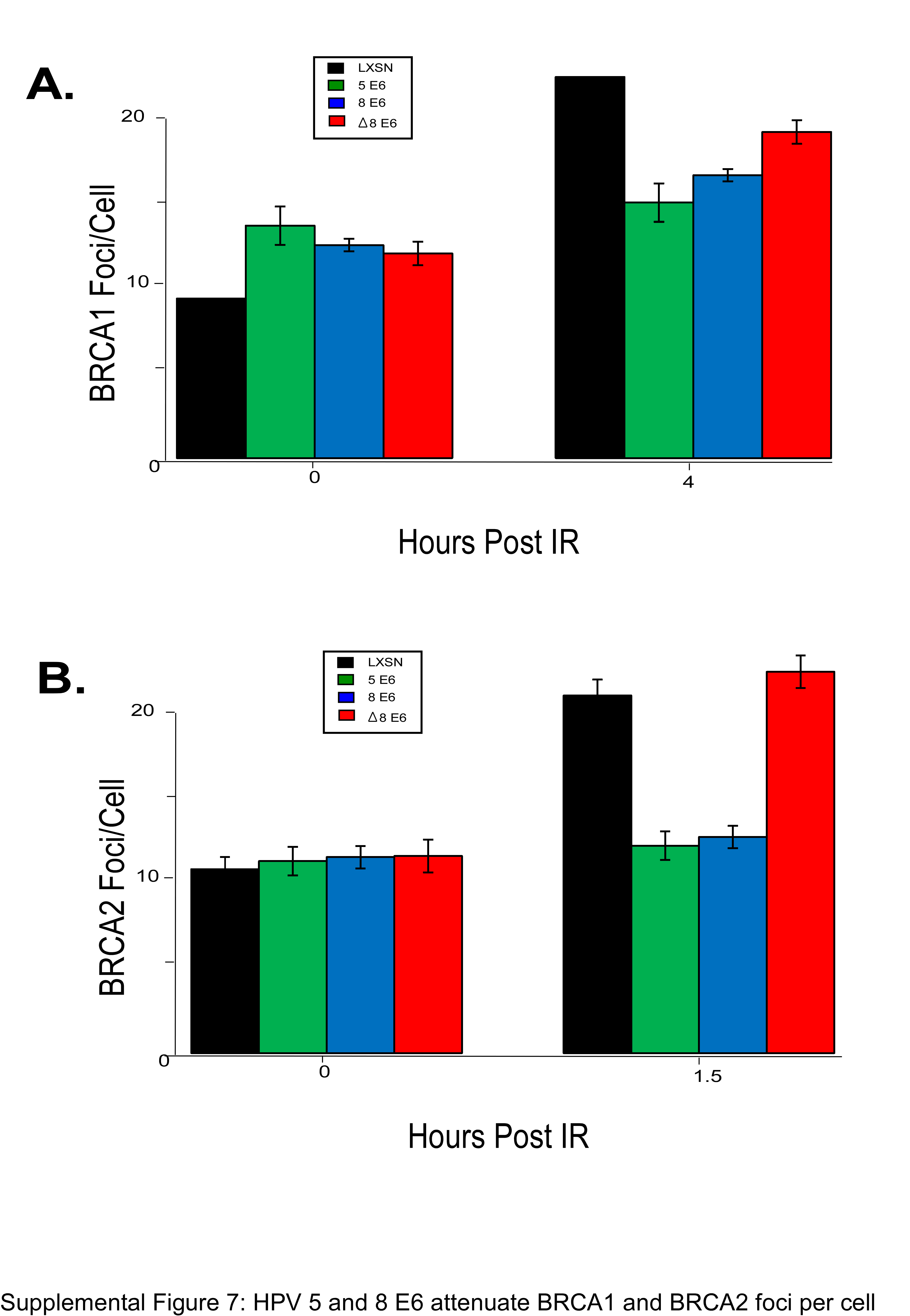

Supplement: S7 Fig — These charts depict the average number of (A.) BRCA1 and (B.) BRCA2 foci per cell following IR exposure. Error bars depict the standard error of the means. n>5 at all points. (TIF) [file ppat.1004687.s007.tif]

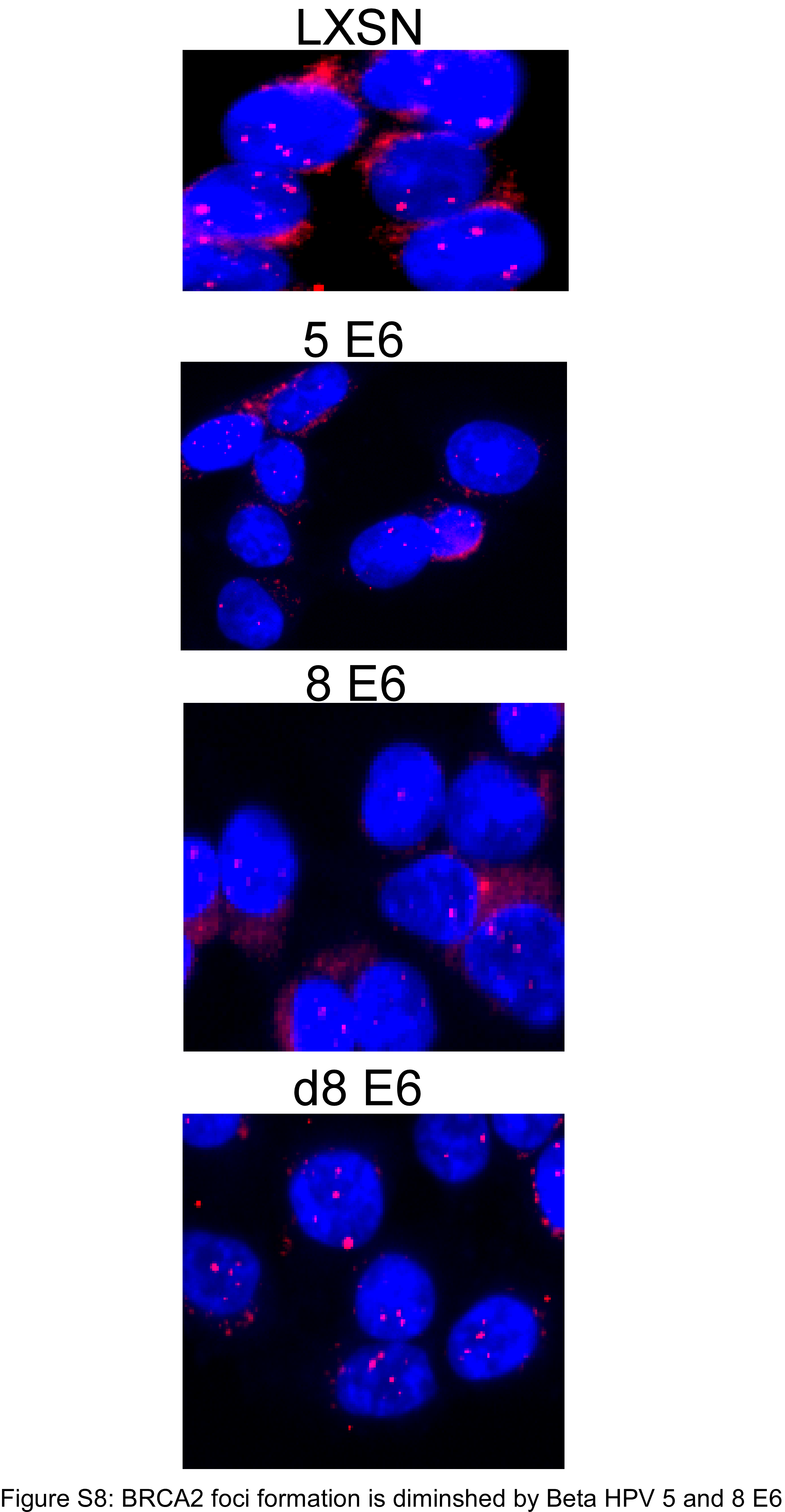

Supplement: S8 Fig — Representative images of cells 90 minutes after exposure to 4 gray of IR with both BRCA2 (pink) and nuclei (blue) staining. (TIF) [file ppat.1004687.s008.tif]

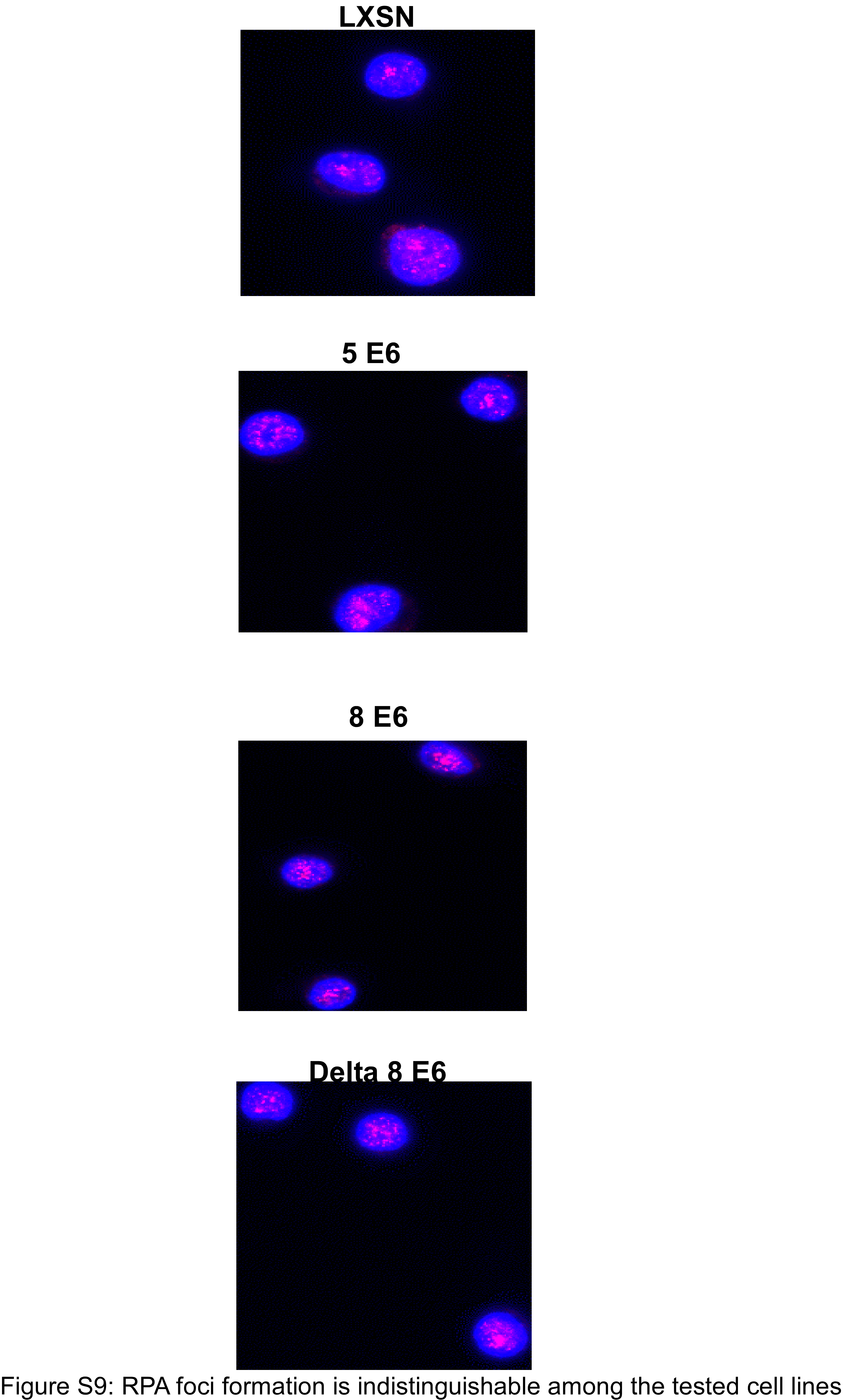

Supplement: S9 Fig — Representative images of cells 90 minutes after exposure to 4 gray of IR with both RPA (pink) and nuclei (blue) staining. (TIF) [file ppat.1004687.s009.tif]

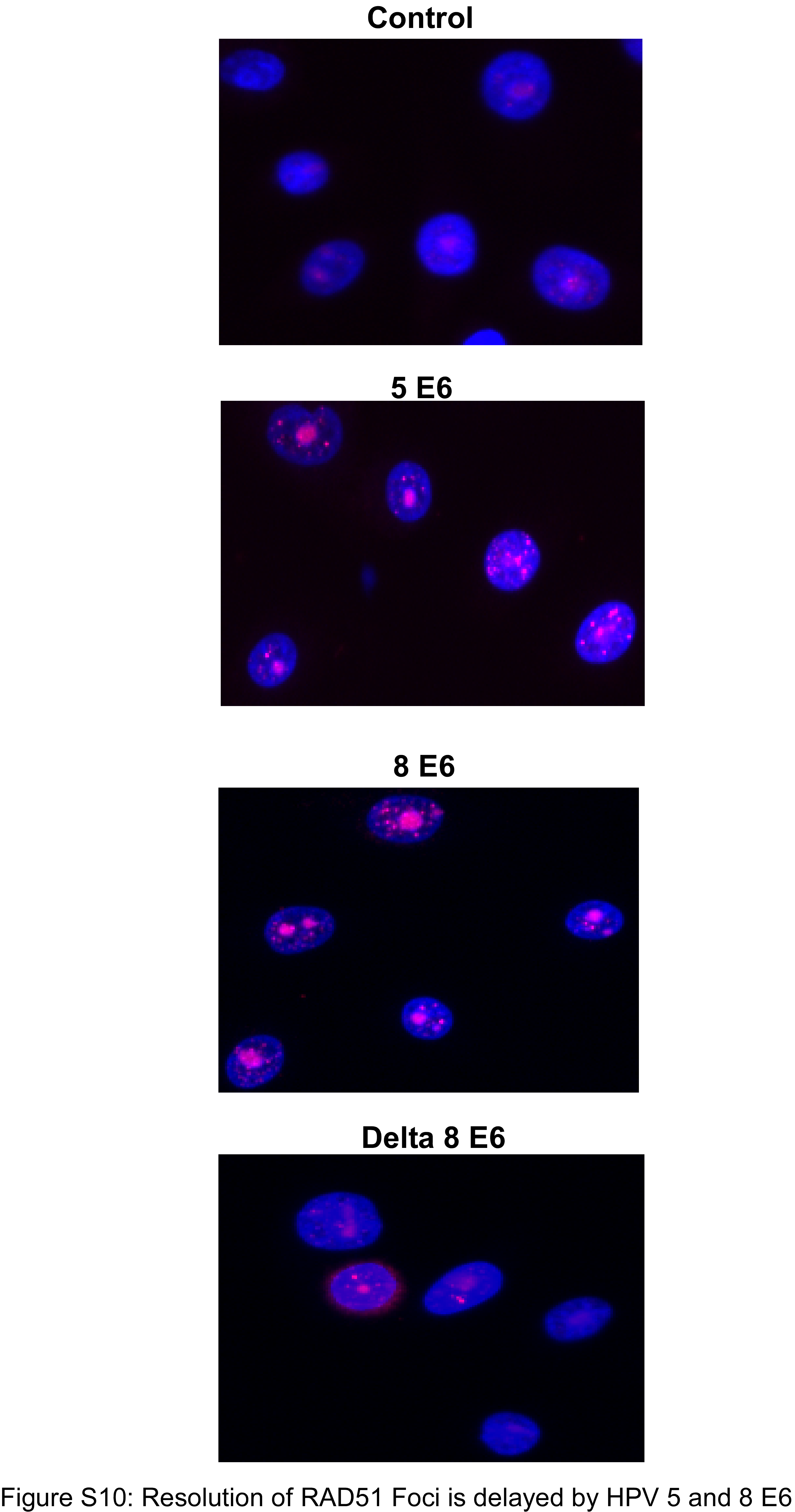

Supplement: S10 Fig — Representative images of cells 24 hours after exposure to 4 gray of IR with both RAD51 (pink) and nuclei (blue) staining. (TIF) [file ppat.1004687.s010.tif]

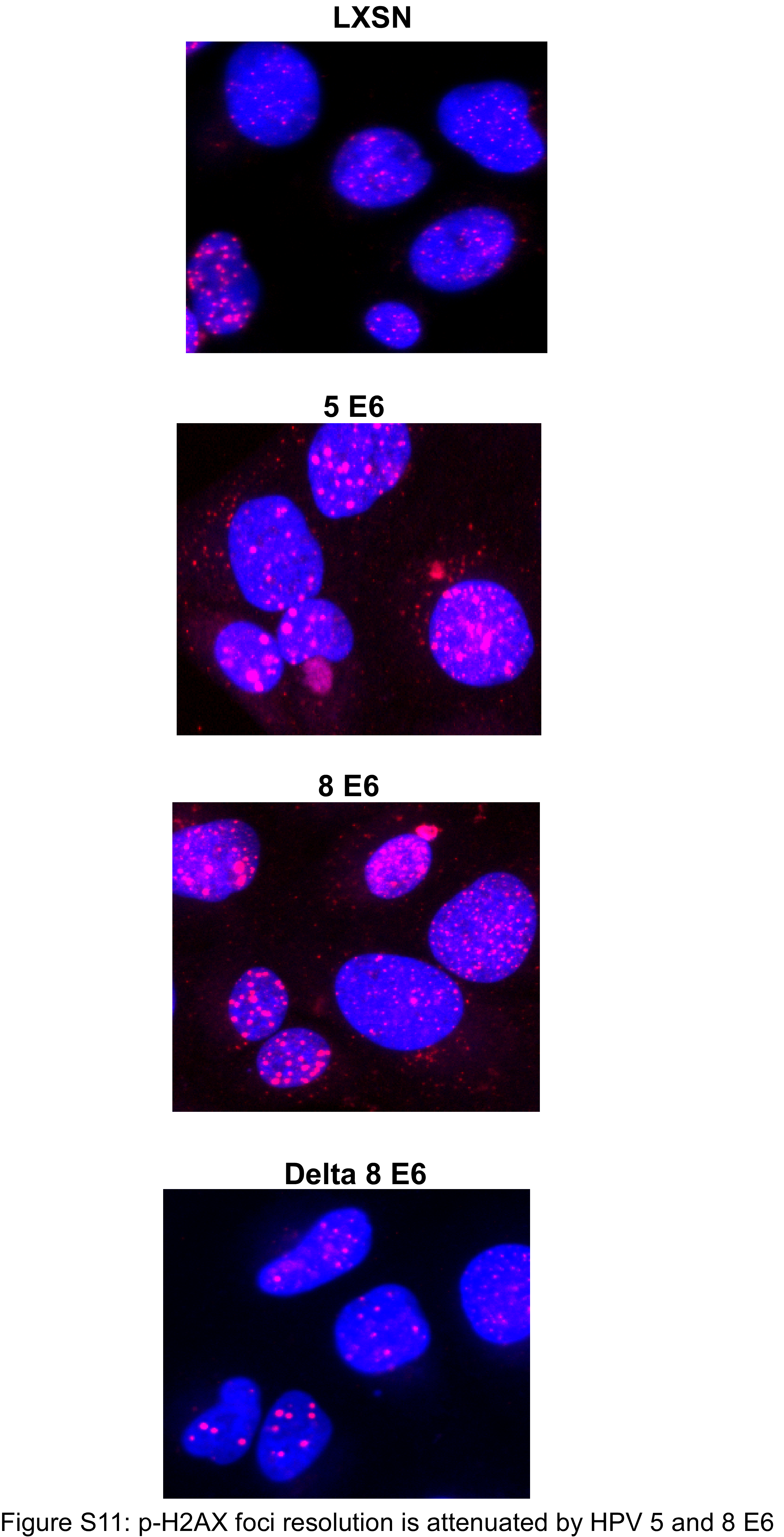

Supplement: S11 Fig — Representative images of cells 6 hours after exposure to 4 gray of IR with both p-H2AX (pink) and nuclei (blue) stained. (TIF) [file ppat.1004687.s011.tif]

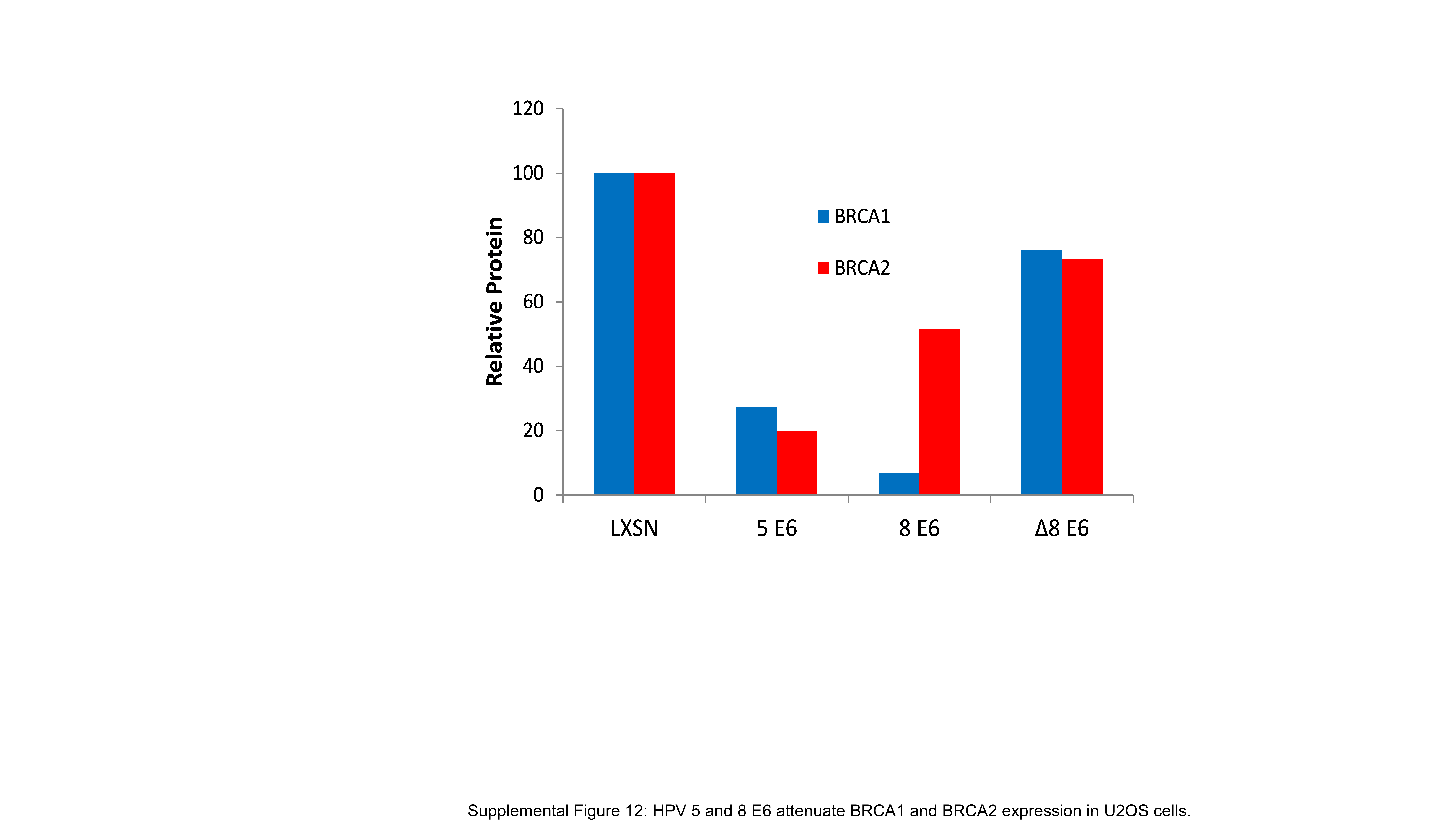

Supplement: S12 Fig — Densitometry of immunoblots of BRCA1 (blue) and BRCA2 (red). The amount of protein in each sample was normalized to the corresponding amount of nucleolin and then data was set relative to vector control (LXSN) cells. (TIF) [file ppat.1004687.s012.tif]

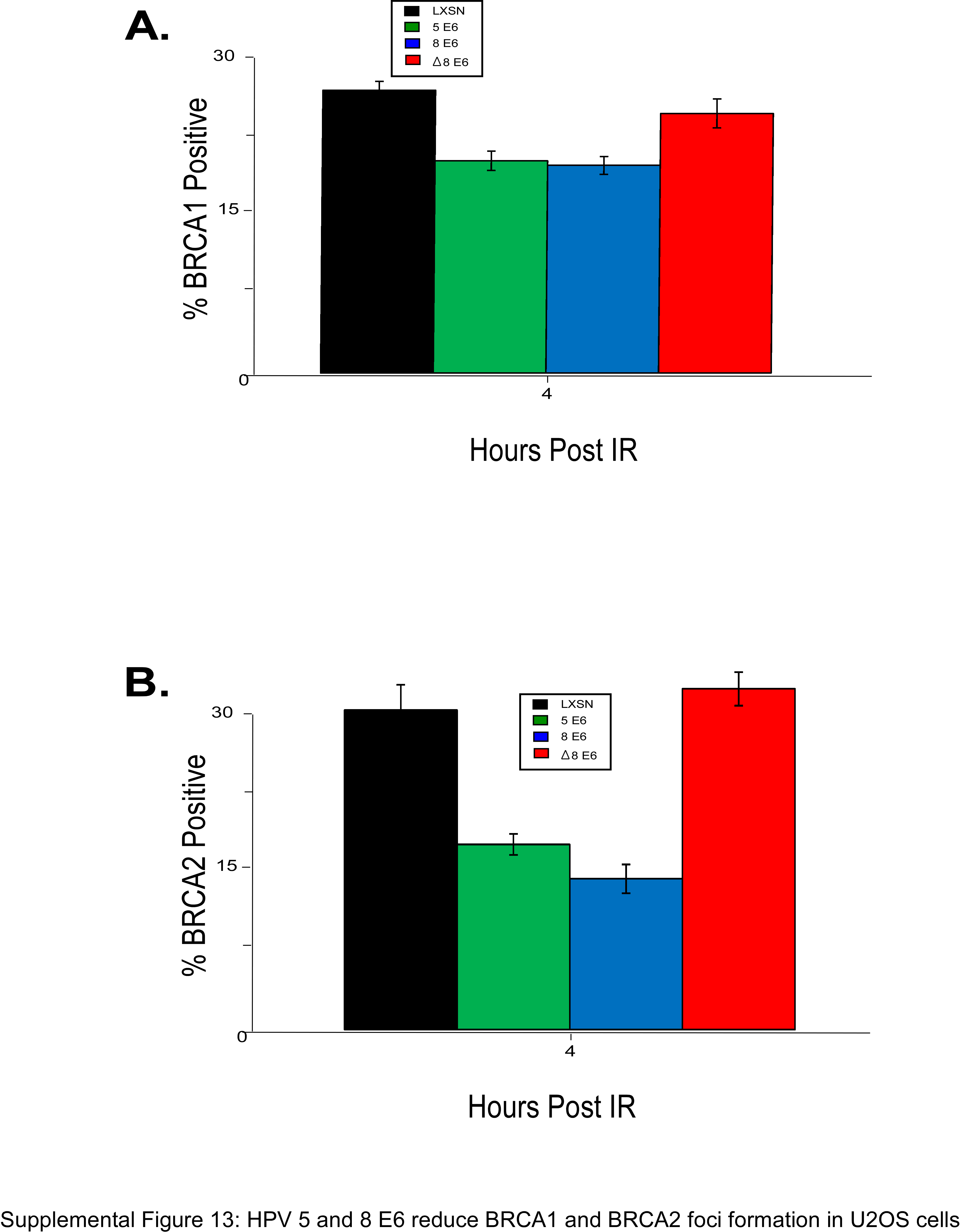

Supplement: S13 Fig — U2OS cells expressing either control, HPV 5 E6, HPV 8 E6 or HPV Δ8 E6 were exposed to 4 gray of ionizing radiation and immunofluorescence microscopy was used to observe (A.) BRCA1 and (B.) BRCA2 foci formation 4 hours after irradiation. (TIF) [file ppat.1004687.s013.tif]

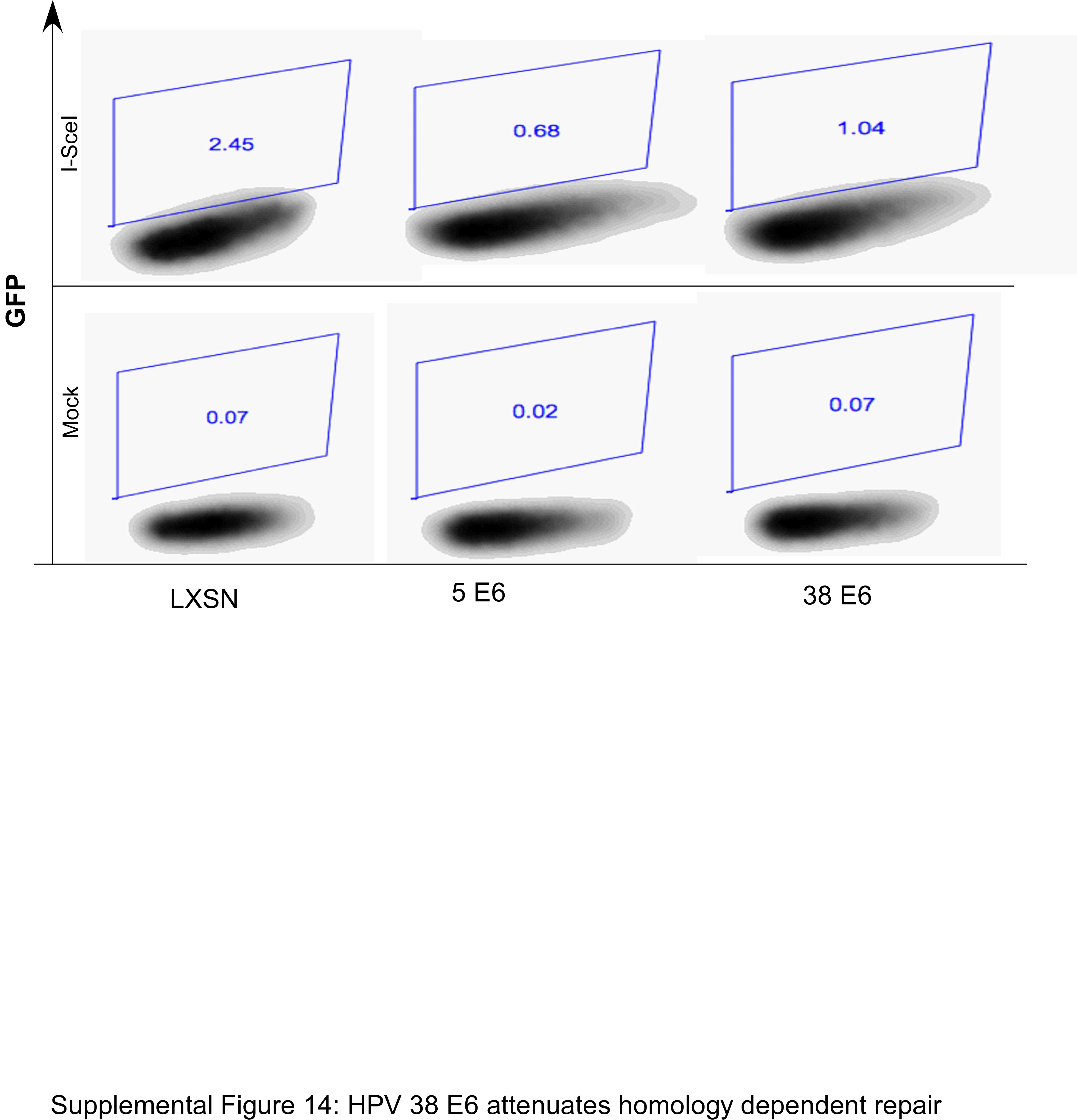

Supplement: S14 Fig — Representative samples of FACS profiles used to generate data shown in Fig. 3C. (TIF) [file ppat.1004687.s014.tif]

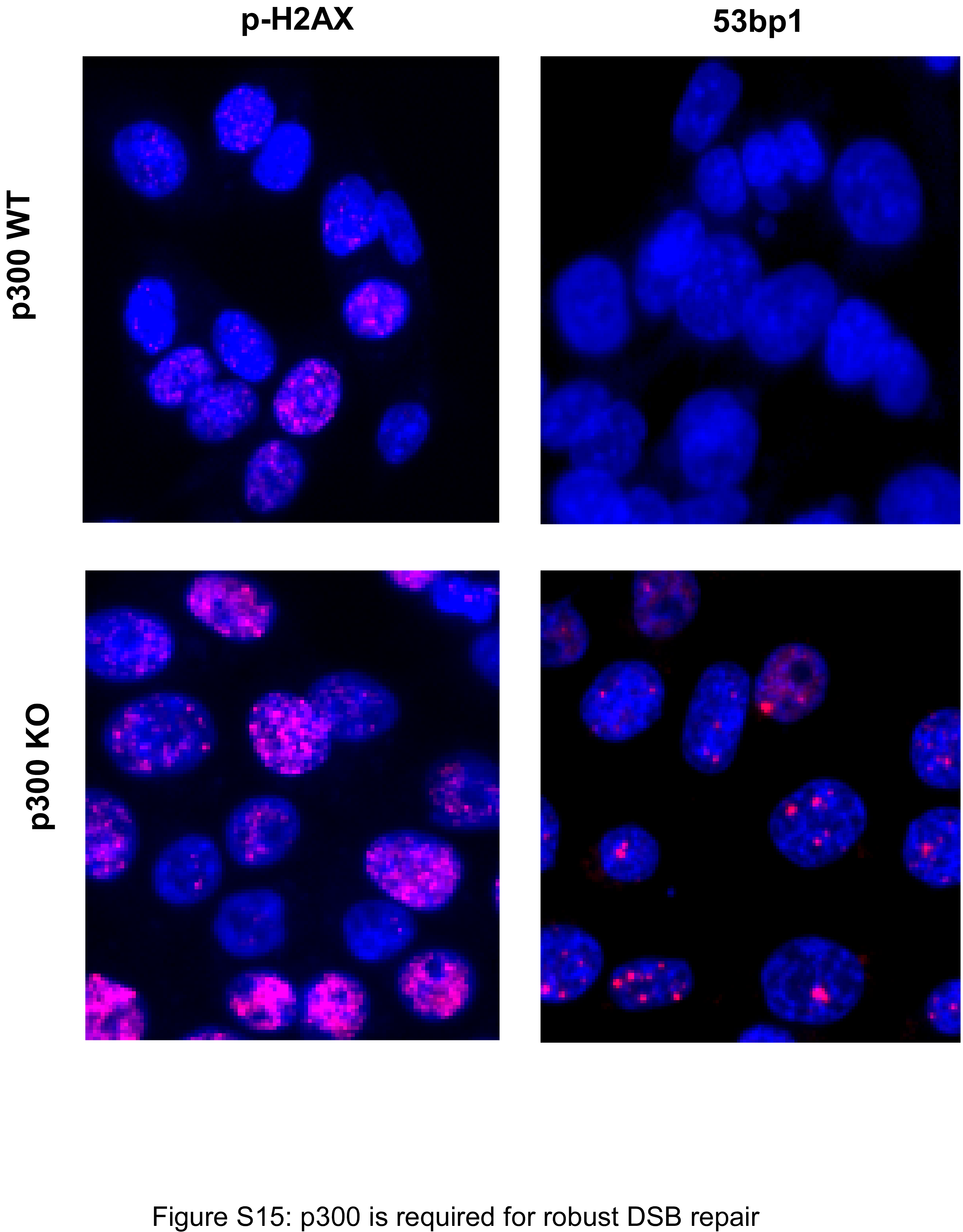

Supplement: S15 Fig — Representative images of cells following exposure to 4 gray of IR with either p-H2AX (pink) or 53bp1 (pink) as indicated and nuclei (blue) stained. (TIF) [file ppat.1004687.s015.tif]

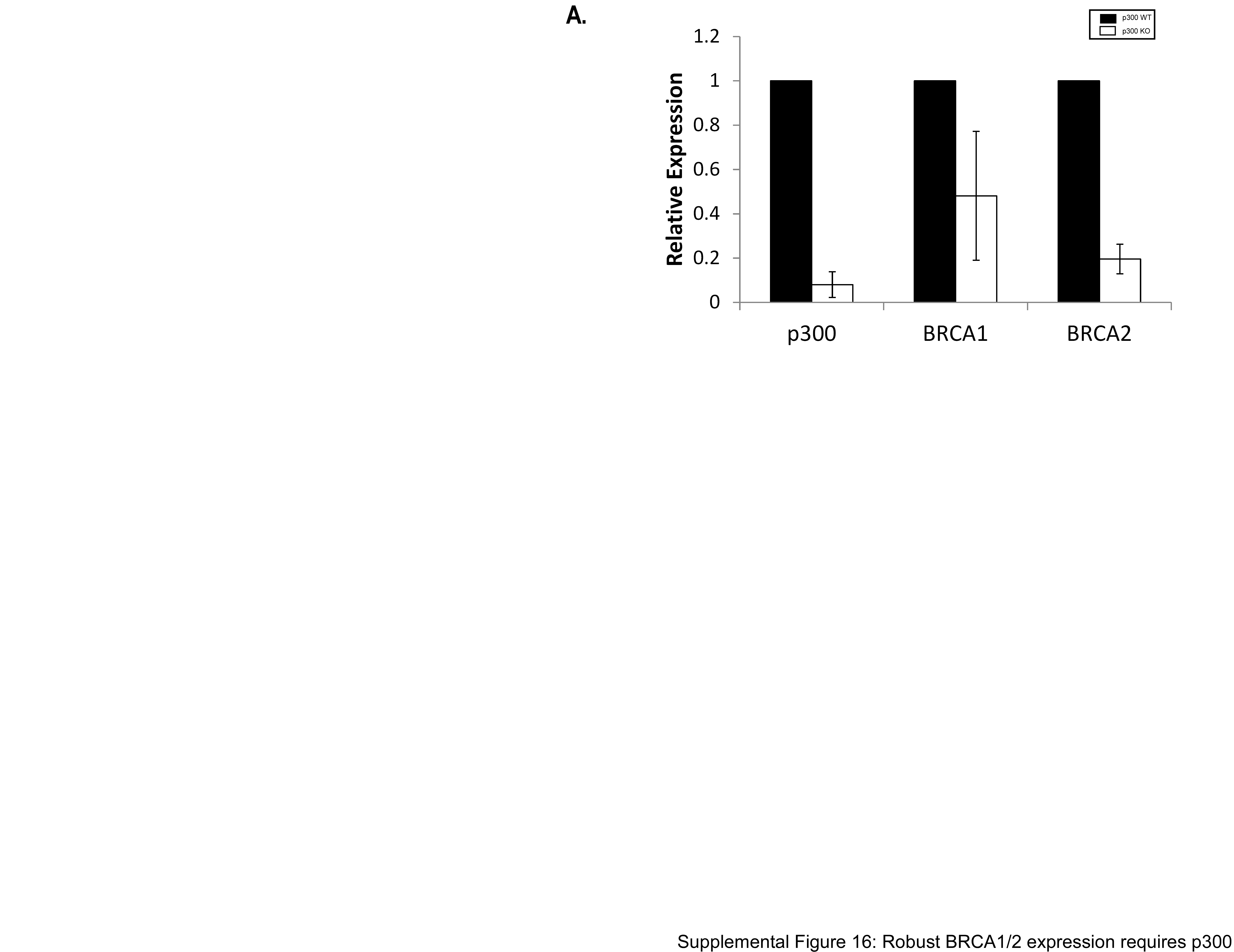

Supplement: S16 Fig — A. Densitometry of immunoblots of BRCA1 and BRCA2 in HCT cells with (black) and without p300 (white). The amount of protein in each sample was normalized to the corresponding amount of nucleolin and then data was set relative to wild type p300 HCT cells. (TIF) [file ppat.1004687.s016.tif]

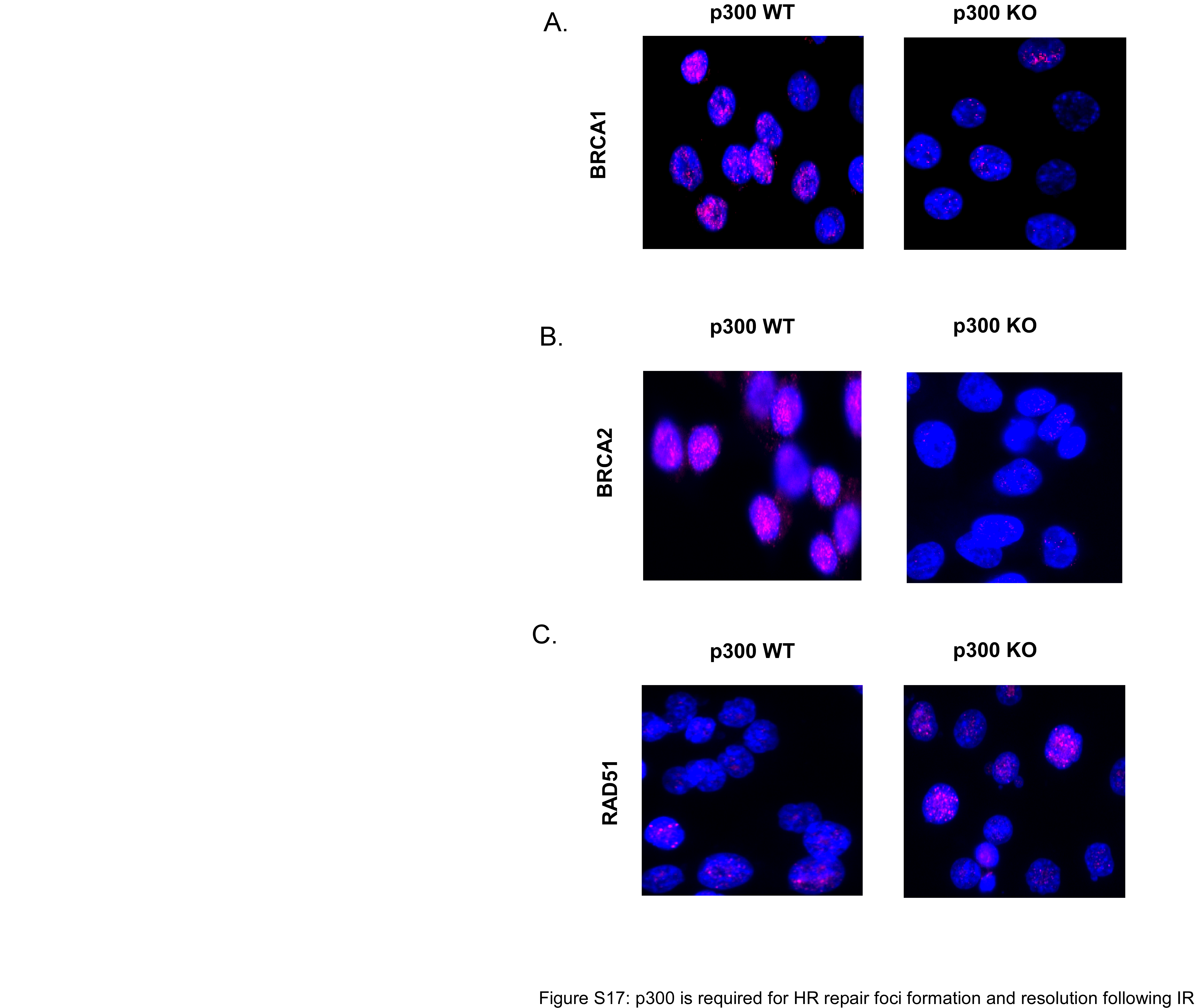

Supplement: S17 Fig — Representative images of cells after exposure to 4 gray of IR with either A. BRCA1 (pink) and nuclei (blue) stained, (B.) BRCA2 (pink) and nuclei (blue) stained, or (C.) RAD51 (pink) and nuclei (blue) stained. (TIF) [file ppat.1004687.s017.tif]

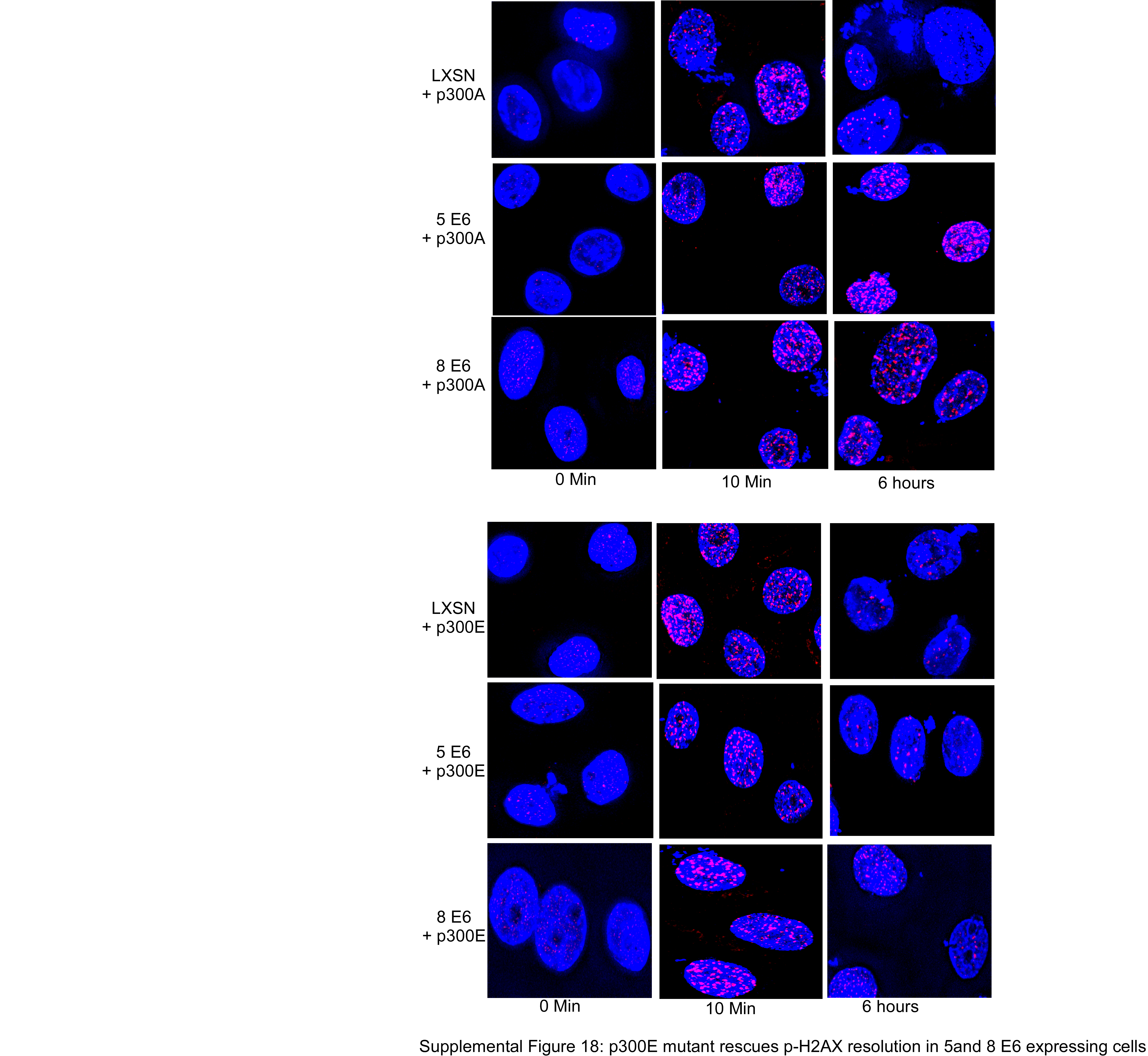

Supplement: S18 Fig — Control, 5 E6, or 8 E6 expressing cells transfected with p300A or p300E as indicated before being stained for p-H2AX (pink) and nuclei (blue) cells 0 minutes, 10 minutes or 6 hours after exposure to 4 gray of IR. (TIF) [file ppat.1004687.s018.tif]

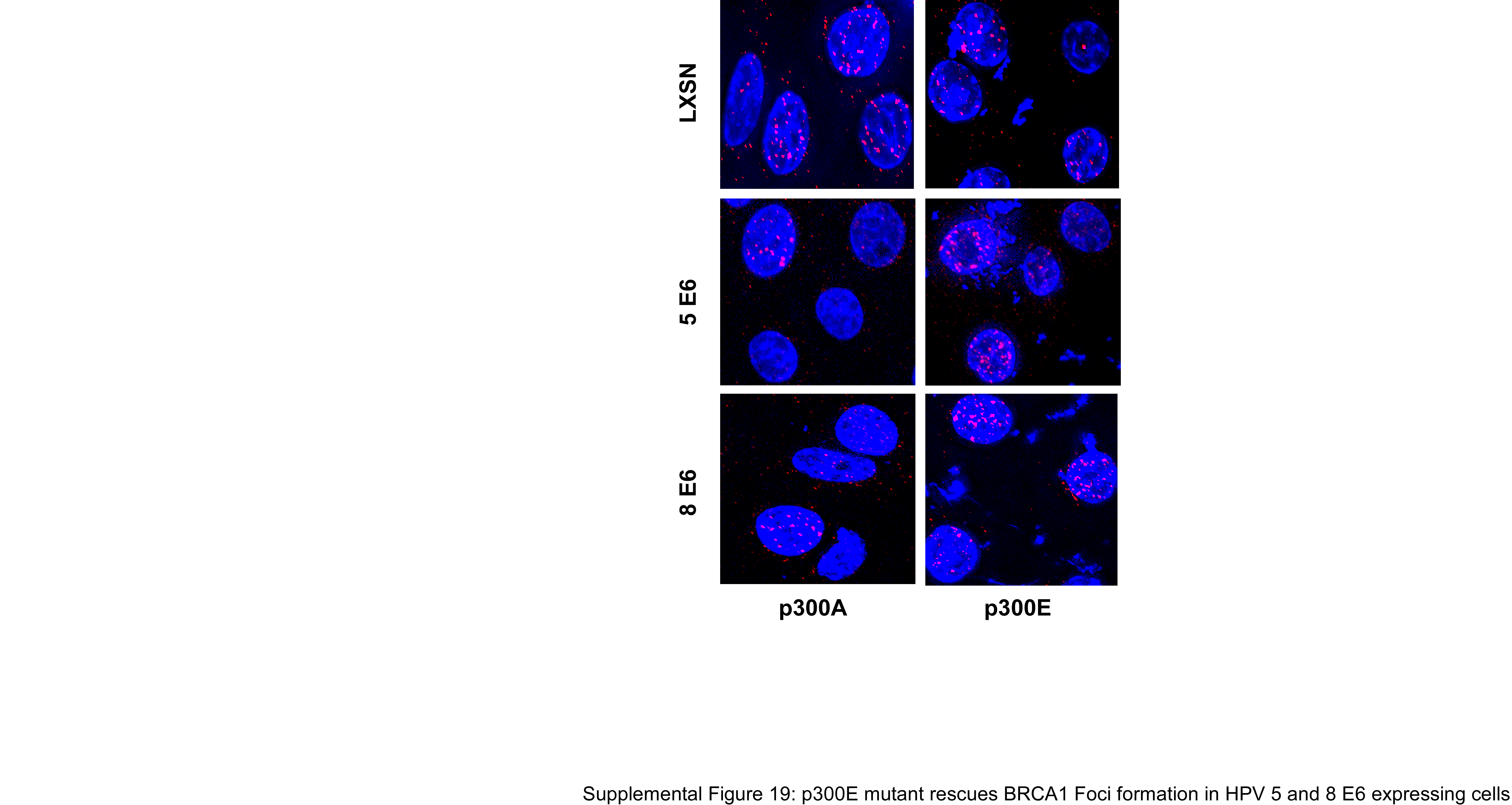

Supplement: S19 Fig — Control, 5 E6, or 8 E6 expressing cells transfected with p300A or p300E as indicated before being stained for BRCA1 (pink) and nuclei (blue) cells 4 hours after exposure to 4 gray of IR. (TIF) [file ppat.1004687.s019.tif]

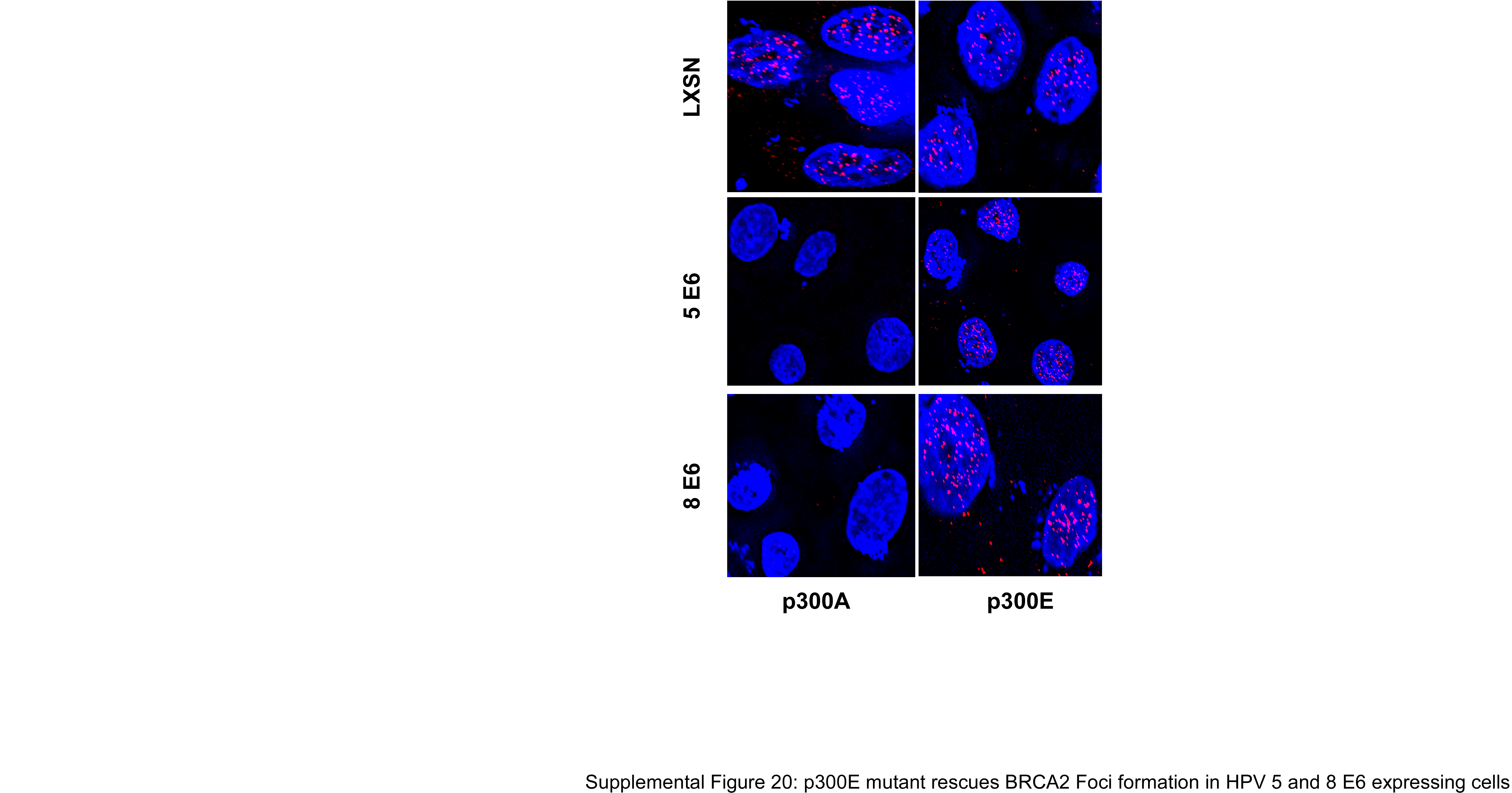

Supplement: S20 Fig — Control, 5 E6, or 8 E6 expressing cells transfected with p300A or p300E as indicated before being stained for BRCA2 (pink) and nuclei (blue) cells 1 hour after exposure to 4 gray of IR. (TIF) [file ppat.1004687.s020.tif]

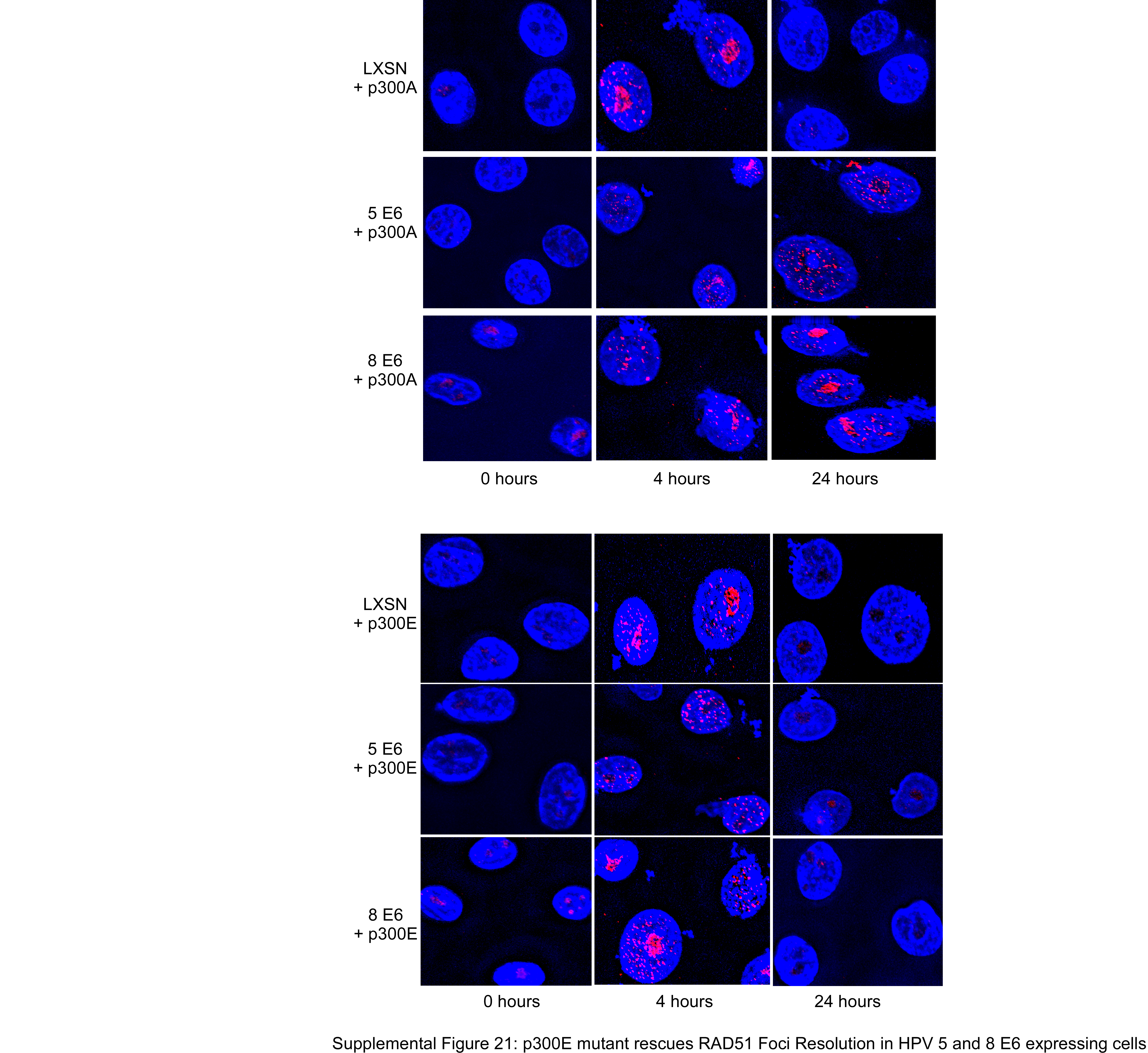

Supplement: S21 Fig — Control, 5 E6, or 8 E6 expressing cells transfected with p300A or p300E as indicated before being stained for RAD51 (pink) and nuclei (blue) cells 0 minutes, 4 or 24 hours after exposure to 4 gray of IR. (TIF) [file ppat.1004687.s021.tif]
